# Understanding bidirectional and transactional relations in parent and offspring mental health: Using COVID‐19 pandemic data to gain insights

**DOI:** 10.1002/jcv2.70057

**Published:** 2025-10-21

**Authors:** Martha Oakes, Lowrie Hilladakis, Polly Waite, Cathy Creswell, Simona Skripkauskaite

**Affiliations:** ^1^ Department of Experimental Psychology University of Oxford Oxford UK; ^2^ Department of Psychiatry University of Oxford Oxford UK; ^3^ Present address: Department of Bioengineering and Therapeutic Sciences University of California San Francisco California USA

**Keywords:** adolescents, children, mental health, pandemic, parents, RI‐CLPM, wellbeing

## Abstract

**Background:**

Bidirectional and transactional models propose that parents and children have the potential to influence each other's mental health over time. While bidirectional associations have been widely studied, transactional processes involving parent internalising, offspring internalising, and offspring externalising symptoms remain underexplored. The COVID‐19 pandemic was associated with marked changes in parent and offspring mental health, providing an enhanced opportunity to examine these dynamics across developmental stages and gender.

**Methods:**

We examined four waves of survey data from the UK‐based longitudinal COVID‐19: Supporting Parents, Adolescents and Children during Epidemics (Co‐SPACE) study collected between May 2020 and May 2021. Data from 2349 parent‐child dyads (4–16‐year‐old children) were analysed using multi‐group (for age and gender) random intercept cross‐lagged panel modelling.

**Results:**

In the full sample, parent internalising symptoms significantly predicted increases in offspring internalising symptoms over time. Among primary school aged children (4–10 years), effects were parent‐driven, with no evidence that changes in child symptoms predicted parent symptoms. In contrast, among secondary school aged children (11–16 years), we found bidirectional associations between parent internalising and offspring externalising symptoms, and some time‐specific links with internalising symptoms. However, no sustained transactional loops (i.e., indirect effects forming a feedback cycle) were detected. Gender did not moderate any associations.

**Conclusion:**

These findings suggest that parent and offspring mental health symptoms may become more reciprocal as children grow older.

## Key Points

**What's Known**

- Parent and child mental health symptoms are known to influence each other, but few studies have examined transactional dynamics over time.

**What's New**

- We found that parent internalising symptoms predicted internalising symptoms in younger children (4–10 years), with no evidence of child‐driven effects.
- Among adolescents (11–16 years), mental health symptoms showed bidirectional associations with parent internalising symptoms, particularly for externalising symptoms.
- Although these consistent reciprocal effects suggest transactional processes in adolescence, we found no evidence of sustained transactional loops across time points.

**What's Relevant**

- Findings support developmentally tailored mental health interventions. Supporting parents may benefit younger children, while interventions for adolescents should consider mutual influences between parent and child mental health over time.

## Graphical abstract

This study found that parent internalising symptoms predicted internalising symptoms in younger children, with no evidence of child‐driven effects. Among adolescents, mental health symptoms showed bidirectional associations with parent internalising symptoms, particularly for externalising symptoms. Although these consistent reciprocal effects suggest transactional processes in adolescence, we found no evidence of sustained transactional loops across time points.

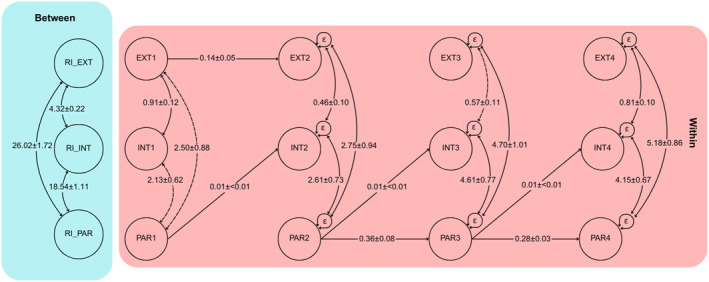

## INTRODUCTION

Bidirectional and transactional models of parent‐child relationships propose that changes in the functioning of either member of a parent‐child dyad have the potential to influence functioning in the other (Bowen, 1978; Sameroff, 1975). Transactional models of development, such as those proposed by Sameroff (1975, 2009), emphasise recursive, dynamic exchanges in which each partner's functioning both affects and is affected by the other over time, creating a feedback loop of mutual adaptation. Bidirectionality may appear when these influences are mutual but does not necessarily unfold over time. Many studies have explored bidirectionality by capturing mutual associations at single or two time points, but fewer have explored transactionality, leaving an important gap in understanding of how ongoing, reciprocal processes may sustain or escalate distress within families. Identifying such feedback loops can help illuminate how parent or child symptoms may reinforce each other over time, pointing to key targets for early intervention and prevention. The COVID‐19 pandemic created a prolonged period of disruption marked by national lockdowns, extended school closures, and social restrictions in the UK between May 2020 and May 2021. Although one large‐scale study suggests that parent‐adolescent relationship quality remained stable during the pandemic (von Soest et al., 2022), further research indicates that adolescent perceived parental support decreased and serious arguments between adolescents and parents increased during the pandemic (Haskell et al., 2025). The potential changes in parent‐offspring relationship quality, alongside fluctuations in both parent and offspring symptoms during this time (Guzman Holst et al., 2023; Skripkauskaite et al., 2023) offer a unique opportunity to examine transactional processes, which may be more difficult to establish when symptom levels are relatively stable.

Studies exploring unidirectional associations provide some insight into how parent and offspring symptoms may influence each other. Studies to date have identified parent‐ and offspring‐driven effects, with some indication that these effects may be moderated by offspring age and gender. Two recent meta‐analyses examining pre‐pandemic studies confirmed significant associations between parent internalising symptoms and offspring internalising and externalising symptoms (Ivanova et al., 2022; Lawrence et al., 2019). For instance, Ivanova et al. (2022) found significant associations from parent depressive to offspring (0–18 years) internalising and externalising symptoms, albeit with small effect sizes. Correlations between parent internalising and offspring internalising and externalising symptoms increased with age across longitudinal studies, while cross‐sectional studies with a higher proportion of girls had larger correlations between parent depression and offspring internalising symptoms and smaller correlations between parent depression and offspring externalising symptoms. Lawrence et al. (2019) found that when parents had an anxiety disorder, their offspring (ranging in age from 3 to 34 years old) were also at higher risk of anxiety or depression disorders. However, this association did not differ across offspring age or gender in this study. While many studies have explored unidirectional effects of offspring mental health symptoms (particularly externalising symptoms) on factors such as parenting style and parent functioning (e.g., see Yan et al., 2021 for a meta‐analyses), fewer studies have explored offspring‐driven effects of internalising and externalising symptoms on parent internalising symptoms. Recent large scale register studies from Denmark, however, suggest that the risk of mental disorder diagnosis is higher among parents whose offspring have a mental health diagnosis (Hakulinen et al., 2025), especially in the years immediately before and after the offspring's diagnosis (Chatwin et al., 2025). Notably, we also identified one study conducted during the COVID‐19 pandemic exploring offspring‐driven effects on parent pandemic‐related stress, which identified small to medium correlations between offspring (4–18 years) externalising symptoms and parent pandemic‐related stress 6 months later (Portnoy et al., 2022).

Consistent with findings from unidirectional studies, studies exploring bidirectional relations have found significant associations between parent internalising and offspring internalising (e.g., Fanti et al., 2013; Hastings et al., 2021; Johnco et al., 2021; Sifaki et al., 2021; Speyer et al., 2022) and externalising symptoms (e.g., Essler et al., 2021; Fanti et al., 2013; Rizeq et al., 2023; Sifaki et al., 2021; Speyer et al., 2022) in children and adolescents. One study explored moderation by offspring (11–18 years) gender and found that maternal depression predicted higher daughters', but not sons', depression, while maternal anxiety predicted higher offspring anxiety independent of offspring gender (Hastings et al., 2021). In contrast, another study found that externalising problems were only positively associated with maternal depressive symptoms across time for girls, but not for boys, while positive bidirectional links between maternal depression and offspring internalising symptoms did not differ according to offspring gender (Fanti et al., 2013). Notably, the above studies explored symptom associations over relatively long follow‐up intervals (≥1 year) so may not have captured the fluctuations and stability in reciprocity that may occur over shorter intervals.

While many studies examining parent and offspring mental health symptom exchange refer to the transactional model, they do not necessarily test the core dynamic processes it implies. Instead, they often explore bidirectional associations at two time points or rely on models that conflate stable between‐person differences with within‐person changes over time. Transactional effects can be operationalised as indirect paths from one individual to the other and back again (i.e., from variables A‐B‐A), yet the majority of studies exploring mental health symptom exchange over time have instead focused on mediation chains involving third variables (i.e., A‐B‐C) (Johnco et al., 2021; Larrucea‐Iruretagoyena & Orue, 2023; Ma et al., 2024; Roubinov et al., 2022). More recent methodological developments, such as the random intercept cross‐lagged panel model (RI‐CLPM), allow for further examination of within‐person fluctuations over time, disentangled from stable, trait‐like differences between individuals (Hamaker et al., 2015; Lucas, 2023). This allows us to test whether deviations from an individual's typical level of symptoms predict subsequent deviations in their partner's symptoms, and vice versa, in line with the theoretical notion of a transactional feedback loop between a parent and child as outlined (Sameroff, 2009). Exploring transactional effects is important since it can depict whether negative loops of influence exist between individuals and in turn indicate whether and via which individuals these loops can be interrupted. For example, if increases in a parent's internalising symptoms consistently precede increases in their child's symptoms, which in turn increase parent's symptoms, early support for the parent could also benefit the child and break the escalation cycle and informing targeted family‐based interventions.

The circumstances of the COVID‐19 pandemic provide an enhanced opportunity to explore how a stressful environment and wider contextual factors may affect associations between parent and offspring mental health symptoms. For example, changes in family circumstances and widespread changes in daily activities (such as lockdowns, home schooling, loss of usual support networks) meant that families often spent more time together with reduced opportunities for contact with others. We are not aware of any studies exploring transactionality among parent internalising and offspring internalising and externalising symptoms during the pandemic or other crises contexts; however, three studies have explored bidirectional links (Black et al., 2021; Essler et al., 2021; Rizeq et al., 2023). Two of these studies explored bidirectional associations between parent internalising and offspring internalising and externalising symptoms over two time points and identified significant bidirectional associations, with small effect sizes (Essler et al., 2021; Rizeq et al., 2023). Essler et al. (2021) explored associations in children (3–11 years) in Germany from April 2020 to July 2020. They found positive effects from child problem behaviour and negative effects from child wellbeing on later parental strain. They also found that reductions in parental strain were associated with improvements in both child wellbeing and problem behaviours. Rizeq et al. (2023) explored associations in children and adolescents (6–18 years) in Canada 40 days apart over two time points during the first 8 months of the pandemic and found positive bidirectional associations between child mental health symptoms and parental mental health symptoms or stress. A third study explored relationships between parent and offspring depression symptoms at one time point by asking about their symptoms retrospectively before the pandemic (using an anchor date of February 2020) and 6 weeks into the pandemic in offspring (8–17 years) in the United States and did not find a significant association in either direction (Black et al., 2021).

To gain insight into the potential bidirectionality and transactionality of mental health symptom exchange between parents and offspring, the current study used parent‐reported data on 4–16‐year‐old offspring's internalising and externalising symptoms and parent self‐reported internalising symptoms from the UK‐based longitudinal Co‐SPACE survey (COVID‐19: Supporting Parents, Adolescents and Children during Epidemics) over four equidistant time points between May 2020 and May 2021. By applying RI‐CLPMs, we examine whether deviations from individuals' typical symptom levels predict changes in their partner's symptoms over time, reflecting the feedback dynamics central to transactional theory. Based on existing theory, we hypothesised that there would be significant concurrent and bidirectional associations between parent internalising and offspring internalising and externalising symptoms, and that associations would be strongest during periods of peak restriction—that is, May 2020 to September 2020; January 2021 to May 2021. Given the absence of previous literature, we set out to explore whether transactional relationships were sustained via indirect relationships over time but made no a priori hypotheses around transactionality. We also sought to investigate whether these potentially bidirectional and transactional relationships differed by offspring age and gender, but, given the mixed findings to date, we did not have a directional hypothesis for either.^1^

## METHODS

### Contextual background

Data were collected between May 2020 and May 2021, a period capturing varying levels of the COVID‐19 restrictions in the UK. Specifically, the UK was under national lockdown during the initial data collection wave (May 2020), with schools closed to most children and strict stay‐at‐home orders. A second national lockdown occurred in January 2021, again accompanied by school closures and restrictions on household mixing. Between these periods (e.g., September 2020), restrictions were partially lifted, though social distancing, limited access to services, and general uncertainty remained prevalent. Restrictions, for most, were fully lifted by May 2021. These fluctuating restrictions offer a unique naturalistic context to examine how environmental stressors may interact with family mental health dynamics.

### Participants

Participants were parents and carers (hereafter known as parents) who took part in the longitudinal UK‐based Co‐SPACE (COVID‐19: Supporting Parents, Adolescents and Children during Epidemics) study (Waite et al., 2025). Parents of school‐aged children (aged 4–16 years at the beginning of the study) were recruited to take part in an online survey through a variety of means, including social media, distribution through partner organisations, networks and charities, the media and targeted advertising.

In total, 9206 parents participated in the Co‐SPACE survey (i.e., reached at least the questionnaire section of the survey) at least once between 30 March 2020 and 31 July 2021. The current study focused on data collected in four equidistant months: May 2020, September 2020, January 2021, and May 2021, selected to represent periods of more stringent (May 2020 and January 2021) and looser (September 2020 and May 2021) social restrictions. A total of 3566 participants who did not complete any surveys during these 4 months were excluded. Additionally, to ensure consistency in the reporting window, only data from follow‐up surveys using the Strengths and Difficulties Questionnaire (SDQ) version referencing symptoms over the past month were included and the baseline SDQ version, which references symptoms over the past 6 months, was excluded. As a result, 1217 participants who completed only the baseline survey during the selected months were excluded. To allow for multi‐group comparisons, 36 participants who either did not report their child's gender or selected ‘Other’ were excluded. Finally, to support estimation of longitudinal effects, 2038 participants who completed fewer than two of the four selected surveys were also excluded. Full demographics of excluded participants can be found in Table S2.

This resulted in a final sample of 2349 participants, with each participant representing one parent‐offspring dyad (see Table 1 for the sample characteristics overall and per wave). The majority of the parents self‐identified as women (2165; 92.2%), with 173 (7.4%) self‐identifying as male, and 11 (0.4%) indicating their gender as ‘Other/Prefer not to say’. Of the offspring in the sample, 1120 (47.7%) were girls and 1229 (52.3%) were boys. 1487 (63.3%) of the offspring were primary school aged (4–10 years old) and 862 (36.7%) were secondary school aged (11–16 years old).

**TABLE 1:** Sample characteristics for the final sample overall and per wave.

	Total sample (*N* = 2349)	Per wave			
		W1: May'20 (*N* = 1378)	W2: Sep'20 (*N* = 1745)	W3: Jan'21 (*N* = 1742)	W4: May'21 (*N* = 1459)
Location					
Greater London	235 (10.0%)	142 (10.3%)	174 (10.0%)	179 (10.3%)	146 (10.0%)
Northern England	459 (19.5%)	255 (18.5%)	350 (20.1%)	337 (19.3%)	282 (19.3%)
Northern Ireland	27 (1.1%)	10 (0.7%)	20 (1.1%)	17 (1.0%)	18 (1.2%)
Scotland	123 (5.2%)	66 (4.8%)	87 (5.0%)	86 (4.9%)	76 (5.2%)
Southern England	1150 (49.0%)	723 (52.5%)	843 (48.3%)	844 (48.5%)	706 (48.4%)
The Midlands	276 (11.7%)	140 (10.2%)	214 (12.3%)	222 (12.7%)	179 (12.3%)
Wales	78 (3.3%)	42 (3.0%)	57 (3.3%)	56 (3.2%)	51 (3.5%)
Missing	1 (0.0%)	0 (0%)	0 (0%)	1 (0.1%)	1 (0.1%)
Parent gender					
Women	2165 (92.2%)	1294 (93.9%)	1627 (93.2%)	1595 (91.6%)	1327 (91.0%)
Men	173 (7.4%)	79 (5.7%)	112 (6.4%)	140 (8.0%)	125 (8.6%)
Other/prefer not to say	11 (0.4%)	5 (0.4%)	6 (0.3%)	7 (0.3%)	7 (0.5%)
Employment status					
Self employed	220 (9.4%)	143 (10.4%)	164 (9.4%)	160 (9.2%)	133 (9.1%)
Unemployed/other	421 (17.9%)	212 (15.4%)	317 (18.2%)	313 (18.0%)	254 (17.4%)
Working full time	771 (32.8%)	471 (34.2%)	566 (32.4%)	561 (32.2%)	492 (33.7%)
Working part time	936 (39.8%)	552 (40.1%)	698 (40.0%)	707 (40.6%)	579 (39.7%)
Missing	1 (0.0%)	0 (0%)	0 (0%)	1 (0.1%)	1 (0.1%)
Household income					
<£16,000	186 (7.9%)	65 (4.7%)	130 (7.4%)	150 (8.6%)	111 (7.6%)
≥£16,000	1997 (85.0%)	1212 (88.0%)	1489 (85.3%)	1464 (84.0%)	1252 (85.8%)
Missing	166 (7.1%)	101 (7.3%)	126 (7.2%)	128 (7.3%)	96 (6.6%)
Parent ethnicity					
Ethnic minorities (excl. white minorities)	104 (4.4%)	41 (3.0%)	79 (4.5%)	88 (5.1%)	70 (4.8%)
White	2225 (94.7%)	1332 (96.7%)	1653 (94.7%)	1636 (93.9%)	1375 (94.2%)
Missing	20 (0.9%)	5 (0.4%)	13 (0.7%)	18 (1.0%)	14 (1.0%)
Child gender					
Girls	1120 (47.7%)	652 (47.3%)	839 (48.1%)	832 (47.8%)	696 (47.7%)
Boys	1229 (52.3%)	726 (52.7%)	906 (51.9%)	910 (52.2%)	763 (52.3%)
Child age					
Primary school aged	1487 (63.3%)	923 (67.0%)	1126 (64.5%)	1097 (63.0%)	953 (65.3%)
Secondary school aged	862 (36.7%)	455 (33.0%)	619 (35.5%)	645 (37.0%)	506 (34.7%)
SEN/ND status					
No SEN/ND	1874 (79.8%)	1119 (81.2%)	1396 (80.0%)	1397 (80.2%)	1167 (80.0%)
SEN/ND	366 (15.6%)	207 (15.0%)	270 (15.5%)	272 (15.6%)	229 (15.7%)
Missing	109 (4.6%)	52 (3.8%)	79 (4.5%)	73 (4.2%)	63 (4.3%)
Child mental health					
Depression/anxiety/other	118 (5.0%)	70 (5.1%)	90 (5.2%)	82 (4.7%)	65 (4.5%)
No	2120 (90.3%)	1254 (91.0%)	1574 (90.2%)	1586 (91.0%)	1330 (91.2%)
Missing	111 (4.7%)	54 (3.9%)	81 (4.6%)	74 (4.2%)	64 (4.4%)

Abbreviation: SEN/ND, special educational needs/neurodevelopmental disorders.

### Procedure

Ethical approval for the Co‐SPACE study was provided by the University of Oxford Medical Sciences Division Ethics Committee (R69060).

All participants provided informed online consent at the beginning of their first (i.e., baseline) survey. Participants took part by completing an online survey hosted on Qualtrics. A link was sent to parents via email once a month after they had completed their baseline survey. The research protocols containing further procedural information are available via the Open Science Framework (OSF; https://osf.io/y8ejg).

### Measures

#### Demographic information

Parents reported on their own and their offspring's age, gender, ethnicity, and total household income (Table 1). Offspring age was dichotomised, with offspring designated into ‘Primary school aged’ (4–10‐year‐olds) and ‘Secondary school aged’ (11–16‐year‐olds) categories. Gender variables were categorised as ‘Boys’ and ‘Girls’, and any other categories were recoded as missing due to the small number of respondents. Household income was categorised into less than £16,000 per year (<£16,000 p.a.) or above or equal £16,000 per year (≥£16,000 p.a.) because this cut‐off reflected an income below 60% of the median income in the UK at the beginning of the study (Department for Work and Pensions, 2023). Parents also reported on whether their offspring had a special educational need and/or diagnosed Attention Deficit Hyperactivity Disorder or Autism Spectrum Disorder. Based on this they were coded as having or not having special educational needs and/or a neurodevelopmental diagnosis (‘SEN/ND’ or ‘no SEN/ND’). Parents also reported whether their child had a diagnosed mental health disorder: answer options included ‘Anxiety’, ‘Depression’, or ‘Other mental health difficulty’ and were coded as ‘Depression/Anxiety/Other’ or ‘No’.

#### Depression, Anxiety and Stress Scale‐21 items

Parent internalising symptoms were self‐reported using the Depression, Anxiety and Stress Scale‐21‐item (DASS) (Lovibond & Lovibond, 1995), which assesses depression, anxiety, and stress symptoms. Participants were asked to score every item on a scale from 0 (‘Did not apply to me at all’) to 3 (‘Applied to me very much or most of the time’). Total DASS score was calculated by adding up the ratings for each item and multiplying the total sum by two. Total scores for the DASS‐21 can range from 0 to 126. Cronbach's *α* for the DASS‐21 total score ranged from .93 to .95 across the four waves (*M* = 0.95, SD = 0.01; Table S3), indicating good internal consistency over time.

#### Strengths and Difficulties Questionnaire

Offspring internalising and externalising symptoms were assessed via the parent‐reported SDQ (Goodman, 1997). The SDQ is a brief behavioural screening questionnaire, which examines five subscales of mental health symptoms in children, including emotional symptoms, conduct problems, and hyperactivity/inattention, peer relationship problems and prosocial behaviour. Each subscale is assessed by five items, which are each rated on a 3‐point scale ranging from 0 (‘Not at all’) to 2 (‘Certainly true’). The maximum total score for each subscale is 10. We used three of the five subscales in our analysis (emotional symptoms, conduct problems and hyperactivity/inattention subscales). The peer relationship problems and prosocial behaviour subscales were excluded owing to potential difficulties reporting on this during periods of social restriction. Internalising symptom scores, therefore, reflect scores from the emotional symptom subscale only, with scores ranging from 0 to 10. Cronbach's *α* for this subscale indicated good internal consistency over time with scores ranging from .81 to .84 across the four waves (*M* = 0.82, SD = 0.02; Table S3). The externalising symptom score reflects the sum of scores for items measuring conduct problems and hyperactivity/inattention. Externalising symptom scores were calculated by adding scores for these subscales, with total scores ranging from 0 to 20. Internal consistency over time was also good for externalising symptom subscale with Cronbach's *α* scores ranging from .83 to .85 across the four waves (*M* = 0.84, SD = 0.01; Table S3). We selected the parent‐reported version of the SDQ, as a self‐report version has been created for and primarily validated for use with adolescents aged 11–17 rather than younger children (Goodman, 2001). The parent‐reported SDQ subscales have been found to have satisfactory psychometric properties in community samples (Goodman, 2001; Stone et al., 2010; Vugteveen et al., 2022).

### Data analysis

We employed four wave RI‐CLPM (Hamaker et al., 2015) to examine the relationships between parent and offspring mental health symptoms, including all three study variables (parent internalising symptoms, offspring internalising symptoms, and offspring externalising symptoms). We selected the RI‐CLPM over the pre‐registered (Table S1) traditional CLPM to account for stable, time‐invariant between‐person differences, which, if unmodelled, can bias within‐person estimates (Lucas, 2023; Rogosa, 1980). This approach is also better suited to detect whether deviations in parent or offspring mental health symptoms predict subsequent deviations in the other's symptoms, thus directly testing the notion of transactional influences. CLPM is conducted and reported as a sensitivity analysis using an alternative approach. The analyses were modelled using Mplus (version 8.7) (Muthén & Muthén, 2017). Nested model selection relied primarily on assessing decreases in Akaike (AIC) and Bayesian information criteria (BIC), as well as chi‐square difference testing (Δ*χ* ^2^) to balance model fit and parsimony (Table S4). Yet, in line with common reporting standards, we also report the robust values of the comparative fit index (CFI ≥ 0.90), Tucker‐Lewis index (TLI ≥ 0.95), root mean square error of approximation (RMSEA ≤ 0.05) and standardised root mean square residual (SRMR ≤ 0.05) as general indicators of absolute model fit (Kline, 2023).

To ensure the integrity of longitudinal comparisons and account for expected changes in variance over time, all analyses were based on unstandardised variable values. The model included covariances between variables at each wave, cross‐lagged paths between variables across waves, and autoregressive stability paths for each variable (see Figure S1 for full graphical representation of the model). We investigated whether residual variances, residual covariances, cross‐lagged paths, and autoregressive paths could be constrained to be equal across waves (i.e., whether these paths were similar or varied between waves). The cross‐lagged paths were then examined to determine the direction of associations between parent internalising symptoms and offspring internalising and externalising symptoms. We interpreted standardised estimates of effect size for cross‐lagged paths as small (*β* = .03), medium (*β* = .05) or large (*β* = .12) (Orth et al., 2024). Standardised estimates are reported in‐text for ease of interpretation, but unstandardised coefficients (*B* ± SE) are presented in figures to enhance transparency and allow evaluation of parameter precision. When significant direct effects were present, potential indirect effects via those paths were also evaluated (e.g., effects of offspring externalising symptoms on later offspring internalising symptoms via parent internalising symptoms) in order to assess potential transactionality.

Moderation analyses via multi‐group invariance testing were used to examine whether there are differences in cross‐lagged paths by offspring age and gender (Mulder & Hamaker, 2021). First, a model with no path constraints across the moderator groups (i.e., offspring age or gender) was modelled, such that all paths were allowed to vary by group in the same model. Second, a model in which cross‐lagged paths were constrained to be equal across groups was created. These models were evaluated and compared using model fit indices (Δ*χ* ^2^, AIC, and BIC). If group constraints were accepted, we then checked whether model paths could be constrained to not vary over time. If group constraints were not accepted, we further iteratively explored whether any paths could be constrained across groups. We explored whether model paths could be constrained to not vary over time within each group but not across them, whether paths could be constrained to not vary over time in one group at the time, and whether only specific paths (e.g., offspring externalising to parent internalising symptoms) could be constrained over time within group(s) (Tables S5 and S6).

For each research variable in any measurement period, on average 33.1% (SD = 7.31; range: 25.7%–42.6%) of observations were missing due to inclusion of participants with two or three (rather than full four) participations only. The pattern of missing values was evaluated using Little's MCAR test (Little, 1988). The test was significant, *χ* ^2^(291) = 369.41, *p* = .001, however, it is well documented that the chi‐squared (*χ* ^2^) test is very sensitive to large sample sizes (Hu & Bentler, 1999). The chi‐squared (*χ* ^2^) degrees of freedom (df) ratio of 1.27 was indicative of a satisfactory fit (Kline, 2023). As such, we used full information maximum likelihood estimation (FIML) with robust standard errors.

## RESULTS

### Descriptive statistics

Means, standard deviations, variances, and covariances for all observed variables for the whole sample, as well as by offspring age and gender, were estimated in the saturated FIML model (Table 2 and Tables S7–S9). Covariance estimates indicated generally moderate to strong positive relationships within and across timepoints, supporting the stability of measured constructs throughout the study period. Raw sample size, means, and standard deviations per wave and per age and gender group are available in Table S10.

**TABLE 2:** Means (*M*) and standard deviations (SD) per wave and by offspring age and gender.

Variable	Total sample		Primary		Secondary		Boys		Girls	
	(*n* = 2349)		(*n* = 1417)		(*n* = 932)		(*n* = 1229)		(*n* = 1120)	
	*M*	SD	*M*	SD	*M*	SD	*M*	SD	*M*	SD
INT1	2.99	2.61	3.09	2.50	2.82	2.77	2.86	2.55	3.12	2.68
INT2	2.65	2.61	2.64	2.51	2.68	2.76	2.54	2.52	2.77	2.70
INT3	2.93	2.60	3.02	2.49	2.79	2.75	2.74	2.55	3.14	2.63
INT4	2.83	2.69	2.87	2.59	2.76	2.84	2.64	2.62	3.04	2.75
EXT1	6.78	4.07	7.37	3.91	5.81	4.16	7.28	4.11	6.21	3.95
EXT2	5.85	4.02	6.22	4.02	5.28	3.96	6.36	4.05	5.29	3.91
EXT3	6.26	4.02	6.71	4.04	5.59	3.90	6.75	3.99	5.73	4.00
EXT4	5.77	4.06	6.16	4.04	5.17	4.03	6.25	4.03	5.26	4.02
PAR1	26.06	19.37	27.31	19.22	24.00	19.42	25.87	18.85	26.28	19.95
PAR2	23.23	21.19	23.62	20.54	22.73	22.20	23.00	20.45	23.42	21.90
PAR3	30.37	24.32	31.57	24.63	28.58	23.72	30.66	24.43	30.17	24.25
PAR4	26.27	23.11	26.32	22.61	26.19	23.71	26.54	22.83	26.03	23.37

*Note*: Estimated from a saturated model using FIML in Mplus. SD computed as the square root of the estimated variances. Numbers 1–4 represent study wave (1 = May'20, 2 = Sep'20, 3 = Jan'21, 4 = May'21).

Abbreviations: EXT, SDQ externalising symptoms; INT, SDQ internalising symptoms; PAR, DASS score; Primary, primary school aged children (4–10 years old); Secondary, secondary school aged children (11–16 years old).

### Main RI‐CLPM

#### Model selection

Full details of model indices for model selection can be found in Table S4. Equality constraints were imposed on cross‐lagged paths to evaluate whether these relations were time‐invariant. Imposing such constraints did not lead to a significantly worse model fit (Δ*χ* ^2^(12) = 11.01, *p* = .529). Hence, all the cross‐lagged paths were constrained to be equal over time in the final model, which showed good model fit (CFI = 0.997, TLI = 0.995, RMSEA = 0.022, SRMR = 0.021). However, model fit deteriorated significantly when imposing equality constraints on autoregressive pathways and residual variances or their covariances, suggesting meaningful time‐specific variation in unexplained variance, and thus these parameters were freely estimated.

#### Direct effects

**FIGURE 1:**
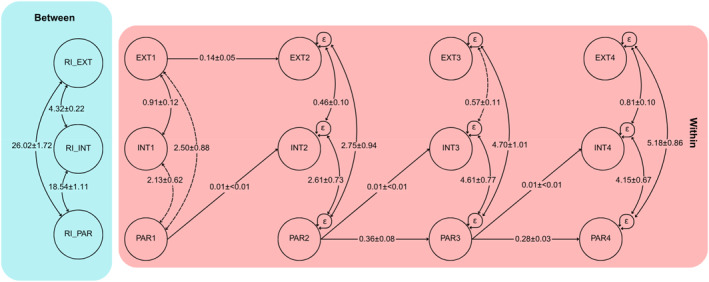
Unstandardised coefficients with standard errors (*B* ± SE) for significant within and between effects in the main RI‐CLPM. EXT, SDQ externalising symptoms; INT, SDQ internalising symptoms; PAR, DASS score; RI‐CLPM, random intercept cross‐lagged panel model. Numbers 1–4 represent study wave (1 = May'20, 2 = Sep'20, 3 = Jan'21, 4 = May'21). Residual variances (*ε*): INT—*ε*_2_ = 1.58 ± 0.12, *ε*_3_ = 1.66 ± 0.14, *ε*_4_ = 2.03 ± 0.11; EXT—*ε*_2_ = 2.60 ± 0.22, *ε*_3_ = 2.63 ± 0.27, *ε*_4_ = 3.14 ± 0.21; PAR—*ε*_2_ = 97.39 ± 15.99, *ε*_3_ = 237.74 ± 10.39, *ε*_4_ = 178.85 ± 8.81.

The unstandardised estimates (*B* ± SE) for significant within and between effects in the RI‐CLPM are displayed in Figure 1 (see Table S11 for full model estimates and standardised coefficients). At the between‐individual level, the random intercepts of offspring internalising, offspring externalising, and parent internalising symptoms were positively and strongly related (*β*: .393–.527). This indicates that parents who reported higher levels internalising symptoms for themselves also reported higher levels of internalising and externalising symptoms for their offspring. Only parent driven longitudinal effects were present at the within‐individual level, however. Higher parent internalising symptoms significantly predicted higher subsequent offspring internalising symptoms with medium effects (*β*: .049–.081) but not offspring externalising symptom scores.

### Moderation by age

#### Model selection

We examined whether longitudinal associations between parent internalising symptoms, offspring internalising symptoms and offspring externalising symptoms differed according to offspring age. In general, we found that they varied between primary school aged (4–10 years) and secondary school aged offspring (11–16 years) as within and between group constraints of cross‐lagged pathways could not be fully imposed, Δ*χ* ^2^(30) = 61.93, *p* < .001 (see Table S5, for further model selection information).

#### Age‐moderated direct effects

**FIGURE 2:**
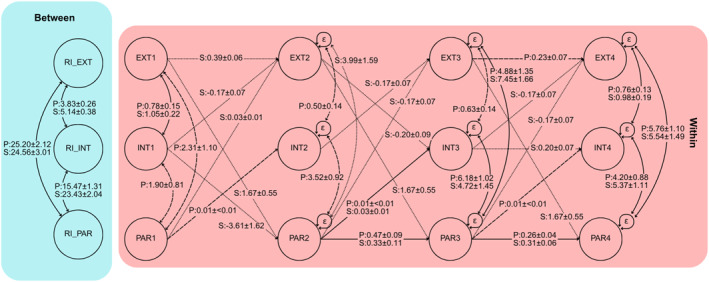
Unstandardised coefficients with standard errors (*B* ± SE) for significant within and between effects in the multi‐group RI‐CLPM for primary (P) and secondary (S) school aged children. EXT, SDQ externalising symptoms; INT, SDQ internalising symptoms; PAR, DASS score; RI‐CLPM, random intercept cross‐lagged panel model. Numbers 1–4 represent study wave (1 = May'20, 2 = Sep'20, 3 = Jan'21, 4 = May'21). Dashed lines—only significant in primary school aged group (P) and dotted lines—only significant in the secondary school aged group (S). Residual variances (*ε*): INT—P: *ε*_2_ = 1.63 ± 0.16, *ε*_3_ = 1.65 ± 0.17, *ε*_4_ = 2.24 ± 0.16; S: *ε*_2_ = 1.10 ± 0.35, *ε*_3_ = 1.65 ± 0.23, *ε*_4_ = 1.79 ± 0.16; EXT—P: *ε*_2_ = 2.88 ± 0.33, *ε*_3_ = 3.30 ± 0.32, *ε*_4_ = 3.26 ± 0.25; S: *ε*_2_ = 2.27 ± 0.24, *ε*_3_ = 1.52 ± 0.39, *ε*_4_ = 2.92 ± 0.43; PAR—P: *ε*_2_ = 106.10 ± 18.82, *ε*_3_ = 259.47 ± 13.84, *ε*_4_ = 178.15 ± 11.23; S: *ε*_2_ = 98.93 ± 36.98, *ε*_3_ = 206.02 ± 14.88, *ε*_4_ = 172.46 ± 13.48.

For primary school aged children (Figure 2), all cross‐lagged pathways could be constrained to be time‐invariant, indicating stable associations between offspring internalising symptoms, offspring externalising symptoms, and parent internalising symptoms over time. We found that, similarly to the main model, parent internalising symptoms predicted higher subsequent offspring internalising (medium to large effects; *β*: .088–.147) but not externalising symptom scores. Offspring symptoms did not significantly predict parent symptoms over time.

For secondary school aged children (Figure 2), only bidirectional paths between parent internalising and offspring externalising symptoms and unidirectional links from offspring internalising to externalising symptoms could be constrained to be time‐invariant. In contrast to the main or primary school aged models, constraining any of the cross‐lagged paths between parent internalising and offspring internalising symptoms to be time‐invariant led to significantly worse model fit (Table S5), suggesting that these relationships changed between waves. There was a significant bidirectional relationship between parent internalising symptoms and offspring externalising symptoms at all time points with large effect sizes (*β*: .133–.320) but only in secondary school aged children. The significant effect between parent and offspring internalising symptoms found in primary school aged children only existed at two cross‐lagged time periods in the secondary school aged group. Specifically, higher offspring internalising symptoms in May 2020 predicted decreases in parent internalising symptoms in September 2020 (*β* = −.257), but higher parent internalising symptoms in September 2020 predicted increases in offspring internalising symptoms in January 2021 (*β* = .231). Full model estimates by age group are presented in Table S12.

#### Age‐moderated indirect effects

We explored whether any indirect pathways were involved in associations between parent and offspring symptoms. Specifically, we tested whether the significant bidirectional relationships between parent internalising and offspring externalising symptoms in secondary school aged group formed a sustained transactional loop. Yet, none of the indirect paths reached statistical significance (*β*: .025–.049, *p* *=* .253). Full details of other direct and indirect pathways in age‐moderated model can be found in Table S12.

### Moderation by gender

Associations between parent internalising, offspring internalising and offspring externalising symptoms were not significantly moderated by offspring gender (see Table S6). The group‐constrained model did not significantly reduce model fit in comparison to the unconstrained model in which all parameters could vary between groups (Δ*χ* ^2^(30) = 36.90, *p* = .180), suggesting that none of the cross‐lagged pathways varied by gender.

### Sensitivity analysis

Sensitivity analyses were performed using a traditional, autoregressive cross‐lagged panel model (CLPM), in line with the original pre‐registration. In summary, both CLPM and RI‐CLPM showed that longitudinal pathways were moderated by offspring age group but not gender, and both identified significant bidirectional effects in the secondary school aged group. However, the specific symptom domains involved in these bidirectional effects differed between models, and the CLPM identified some additional pathways that were not significant in RI‐CLPM. See Supporting Information (Appendix S1; Tables S13–S17) for a full account of this analysis.

## DISCUSSION

The current study examined the reciprocal dynamics between parent and offspring mental health between May 2020 and May 2021 during the COVID‐19 pandemic. We found that the nature and direction of symptom exchange varied by the offspring age group but not their gender. We found that effects were parent‐driven in primary school aged children (4–10 years). In contrast, in secondary school aged children (11–16 years), consistent bidirectional relationships emerged between parent and offspring mental health, particularly involving offspring externalising symptoms. Whilst this bidirectionality may indicate short‐term transactional relations between parent internalising and offspring externalising symptoms in the secondary school aged children, there was no evidence of sustained transactionality over time. Nevertheless, these findings suggest that family mental health processes become more reciprocal as children grow older, pointing to critical windows for intervention that consider both age and symptom type.

Our finding that there was no evidence of offspring‐driven effects (and in turn no evidence of transactionality) in primary school aged children suggests that younger children's and parent's mental health symptoms may not yet form a reciprocal loop over time, particularly during stressful periods. The finding that effects in this age group were uniquely parent‐driven aligns with wider literature on intergenerational transmission, which show that parents' mental health can shape offspring's emotional and behavioural development through both genetic predispositions (Akingbuwa et al., 2022; Eilertsen et al., 2022) and environmental mechanisms, including modelling, parenting behaviours, and emotional climate (Goodman & Gotlib, 1999; Stein et al., 2014). Furthermore, there may be differences in how parents appraise and respond to younger children's behaviours and emotions in comparison to older children. For example, Dix et al. (2012) proposed that parents may appraise negative behaviours as being more challenging and potentially harmful in older children who may be sufficiently developmentally mature to control their behaviours. They also suggest that parents may view older children's behaviour as being less modifiable and in turn potentially more stressful in comparison to younger children where parents may be more likely to perceive their behaviours to still be malleable. Recent evidence from Kochanova et al. (2021) supports this notion, where it was proposed that parents may feel less distressed by their child's difficulties if they consider them normal or excusable for the child's age group. Qualitative findings from the Co‐SPACE study support this explanation, as parents reported that they were aware that their younger child's behavioural or emotional difficulties might be linked to their developmental stage during the pandemic (Shum et al., 2023).

While we only found evidence of parent‐driven effects among the primary school age group, consistent with wider literature (Ivanova et al., 2022; Lawrence et al., 2019), we found bidirectional effects between parent internalising and offspring externalising symptoms in secondary school aged children, suggesting that there is exchange between parent and older offspring's mental health symptoms over time. This finding is consistent with correlational findings from a meta‐analysis by Ivanova et al. (2022) where associations between parent internalising and offspring internalising symptoms were greater with age. Combined with our finding of stable parent‐driven effects in primary school aged children, these findings are consistent with intergenerational transmission (Goodman & Gotlib, 1999) and transactional (Sameroff, 2009) theories suggesting that early transmission is more heavily shaped by caregiving and family environment, with reciprocity becoming more likely as children develop greater cognitive and emotional autonomy. As offspring become more emotionally expressive and autonomous in adolescence, their mental health can more readily affect parents, creating feedback loops that reinforce or exacerbate family‐wide mental health difficulties. Notably, however, this association did not form a sustained reciprocal loop over time via substantial indirect relationships, suggesting that consistency of such symptom exchange may be more pronounced short term (within the 4‐month time lag) rather than forming ongoing, self‐perpetuating feedback loops. Hence, these findings emphasise the importance of timely interventions that are more targeted, time‐sensitive, and adaptable, focusing on the moments when symptoms are reactive rather than assuming a chronic, escalating cycle, especially as children develop greater emotional autonomy. While we used age groupings to examine broad developmental differences in these patterns, future research should also explore whether symptom reactivity and recovery vary more gradually with age by modelling age as a continuous moderator.

We expected that relationships between parent and offspring symptoms would vary over time and be strongest during periods of peak restriction, but this was only partially confirmed for time‐sensitive relations between parent and offspring internalising symptoms in the secondary school aged group. Specifically, we found evidence of offspring‐driven effects early in the pandemic, where higher offspring symptoms in May 2020, when restrictions were stringent, strongly predicted a decrease in parent symptoms in September 2020, when restrictions were loosened. When a second wave of restrictions was introduced, this pattern reversed to parent‐driven effects, with higher parent internalising symptoms in September 2020 strongly predicting an increase in offspring internalising symptoms in January 2021. However, neither bidirectional relationships involving offspring externalising symptoms in secondary school aged children, nor parent‐driven effects on offspring internalising symptoms in primary school aged children, showed meaningful variation across restriction periods. These findings should be considered in the context of a broader literature that shows a mixed picture in terms of the stability of adolescent‐parent relationships during the pandemic (e.g., von Soest et al., 2022; Haskell et al., 2025). Taken together, our findings may suggest that adolescent and parent internalising symptoms are more likely to dynamically influence each other during acute periods of external stress, such as school closures or social isolation. In contrast, the more stable reciprocal relationships observed between parental internalising and adolescent externalising symptoms may reflect ongoing family stress dynamics, where adolescents externalising behaviours, both affect and be affected by parent symptoms, regardless of broader contextual changes.

This interpretation is further supported by our model selection results, which indicated that residual variances and covariances could not be constrained across waves pointing to instability not only in associations between symptoms but also in their unexplained variance. In line with Plomin's (2024) ‘gloomy prospect’, these fluctuations likely reflect real but idiosyncratic interchange between biological, psychological, and environmental factors, such as changes in routine, work pressures, or access to schooling, that affected families in diverse ways across time. For instance, the marked increase in residual variance for parent internalising symptoms during January 2021 may reflect the UK's second lockdown, during which schools reclosed abruptly, outdoor activity was limited due to winter weather, and pandemic‐related fatigue had accumulated. These contextual stressors may have contributed to increased variability in parent emotional responses and symptom expression, further underscoring the need to model time‐specific parameters flexibly.

We had also anticipated, based on recent, albeit conflicting, findings from Ivanova et al. (2022) and Fanti et al. (2013), that there would be gender moderation effects in the association between parent internalising and offspring internalising and offspring externalising symptoms, but we did not find a moderating effect in line with Hastings et al. (2021) and Lawrence et al. (2019). In light of the mixed literature on this topic, further studies are needed to understand whether there are particular circumstances in which offspring gender is involved in the association between parent internalising and offspring internalising and externalising symptoms.

### Limitations

This study used rigorous longitudinal methods to examine bidirectional and transactional associations between parent internalising, offspring internalising and offspring externalising symptoms. However, our findings should be understood in the context of some limitations. For instance, we used parent reports of offspring mental health symptoms to enable valid comparisons across the age‐range of interest. This approach was necessary, as the SDQ self‐report version has only been validated for children aged 11–17 years (Goodman, 2001). Nevertheless, parents might under‐report mental health symptoms in adolescent children (Arman et al., 2013), and their reports may be influenced by other factors, such as their own mental health (Curhan et al., 2020; Najman et al., 2000). While our use of RI‐CLPM helps to account for stable, trait‐like reporting tendencies within reporters and therefore reduce the impact of such common method bias (Hamaker et al., 2015; Lucas, 2023; Rogosa, 1980), it does not fully address potential common method variance or rater bias. The use of multiple informants in future research would strengthen conclusions regarding cross‐informant symptom dynamics. Despite the strengths of RI‐CLPM, it should be noted that directionality and symptom specificity of findings may be partially dependent on the modelling approach. To assess the robustness of our findings, we conducted sensitivity analyses using a traditional CLPM, which produced largely comparable patterns of age moderation and adolescent bidirectionality, though some additional pathways and differences in symptom‐specific effects were observed. Further, this study population was a convenience opportunistic sample biased toward middle and high‐income, highly educated families from White British backgrounds primarily in the South of England. These families were more likely to have possessed greater resources (e.g., computers, stable Internet and financial stability) than those who showed consistently elevated mental health difficulties throughout the pandemic (i.e., low‐income, single adult) (Adegboye et al., 2021; Guzman Holst et al., 2023). The relative underrepresentation of low‐income families or single‐adult households in our sample means that our analysis was not able to assess these factors as potential moderators and may not capture the impact of stringent social restrictions on parents and children from families that were most negatively affected by lockdown. Whilst generally comparable, our final sample also may differ from those lost to attrition in ways that were not captured but could influence generalisability. Thus, we encourage caution in interpreting findings as representative of all families initially enrolled in the study or those living in the UK. Finally, while the pandemic context provided an enhanced opportunity to assess changes in offspring and parent mental health symptoms, we cannot evaluate whether the bidirectional relationships reported here reflect a deviation from the levels of reciprocity between parent and offspring mental health that might be seen in other contexts. Studies examining bidirectional or transactional relationships at similar intervals over a similar number of time points during years unaffected by a public health crisis would provide a useful comparison. Future longitudinal work across different time‐lags is needed to understand how such bidirectional dynamics unfold over shorter or longer timescales and how they relate to longer‐term intergenerational pathways of risk transmission.

## CONCLUSION

This study provides nuanced longitudinal evidence of how parent and offspring mental health symptoms interrelate with one another over time, with clear developmental differences between primary and secondary school‐aged children. We found that parent‐driven effects were most apparent in younger children, while secondary school aged children showed bidirectional exchange with short transactional loops, especially in relation to externalising symptoms. These patterns suggest that the parent–offspring symptom interplay may shift with age, potentially reflecting developmental changes in autonomy, emotional expression, and family stress dynamics. Although some associations appeared sensitive to the broader context of the pandemic restrictions, many effects were consistent across time, underscoring the persistence of family‐based mental health transmission. These findings highlight the need for developmentally tailored mental health interventions that support both parents and children, particularly during times of external stress.

## AUTHOR CONTRIBUTIONS

**Martha Oakes**: Data curation; formal analysis; project administration; visualization; writing—original draft. **Lowrie Hilladakis**: Conceptualization; data curation; formal analysis; methodology; project administration; supervision; writing—review and editing. **Polly Waite**: Funding acquisition; investigation; project administration; supervision; writing—review and editing. **Cathy Creswell**: Funding acquisition; investigation; project administration; supervision; writing—review and editing. **Simona Skripkauskaite**: Conceptualization; data curation; funding acquisition; investigation; methodology; project administration; supervision; validation; writing—review and editing.

## CONFLICT OF INTEREST STATEMENT

The authors declare no conflicts of interest.

## ETHICAL CONSIDERATIONS

Ethical approval was provided by the University of Oxford Medical Sciences Division Ethics Committee (reference number: R69060) on 27 March 2020. Informed consent was appropriately obtained from all participants.

## Supporting information

Supporting Information S1

## Data Availability

Data openly available in a public repository that issues datasets with DOIs. The OSF link is https://osf.io/8zx2y/.

## ENDNOTE

^1^
This study was pre‐registered (https://doi.org/10.17605/OSF.IO/AJEXN) in 2022 with directional hypotheses for the moderating effect of offspring age and gender. However, recently published (pre‐pandemic) studies have mixed findings which highlight the need for a more exploratory approach to the moderation effects rather than leading to clear directional hypotheses. See Table S1 for a full list of deviations from the pre‐registration.
