# Supplementary material for: Understanding bidirectional and transactional relations in parent and offspring mental health: Using COVID‐19 pandemic data to gain insights
Source: JCPP Adv. 2025 Oct 21;6(2):e70057. doi: 10.1002/jcv2.70057 (PMC13260664; doi:10.1002/jcv2.70057)
Supplement: Supplementary file 1 — Supporting Information S1 [file JCV2-6-e70057-s001.docx]

# **Understanding bidirectional and transactional relations in parent and offspring mental health: Using COVID-19 pandemic data to gain insights**

# **Supporting Information**

**Table S1**

*Full list of deviations from the pre-registered analysis plan*

| Section | Pre-registration plan | Current approach | Reason |
| --- | --- | --- | --- |
| Directional Hypotheses | Expected stronger effects for parents with younger than older children (H8); bidirectional relationships varying by child gender (externalising symptoms for boys and internalising symptoms for girls; RQ4). | No directional hypotheses specified; exploratory approach to age and gender moderation. | Recent mixed findings in the literature called for a more exploratory stance. Directional hypotheses were not strongly supported by newer evidence. |
| Indirect / Mediation Pathways | No mention. | Explored indirect pathways without specific hypotheses. | Based on assumptions in the literature about sustained bidirectional links, exploration was deemed valuable even without clear precedent. |
| Sample Size Estimate | Estimated ~3,000 participants. | Final sample: 2,349 dyads. | Slightly below the estimate, but above the minimum threshold (1,500) noted in the pre-registration's ‘Statistical power’ section. |
| Inclusion/ Exclusion Criteria | - ≥2 surveys in different time periods  - Completion of SDQ & DASS  - Exclude impossible values  - One child per parent included | Pre-registered criteria were applied as described.  Additional exclusions:   - Baseline surveys - Missing age/gender info | - Baseline SDQ covered 6 months vs. 1 month at follow-ups, compromising comparability for lagged analysis. - FIML requires no missing data on exogenous moderators; exclusions necessary for proper model estimation. |
| Analytical Method | Cross-lagged panel model (CLPM) | Random Intercept CLPM (RI-CLPM) as primary; CLPM as secondary | The RI-CLPM was included as a supplementary analysis in the pre-print. Peer review emphasized the importance of accounting for stable between-dyad differences and common method bias. |
| Complete Data Sensitivity Analysis | Planned with full 4-wave completers. | Not included. | Included in the pre-print but not the final manuscript:   - RI-CLPM model complexity increased power requirements - Prioritized brevity for manuscript limits |
| Bootstrapping | Planned to assess reliability and robustness. | Applied to all indirect relationships. | Focused on indirect relationships that are particularly likely to not be normally distributed even in large samples. |
| Exploratory Analysis | Re-run models by DASS subscales (if significant relationships found). | Not conducted. | Prioritized main models (RI-CLPM and CLPM); subscale analysis was deprioritized for clarity and space reasons. |

**Table S2**

*Demographic characteristics for excluded participants and final sample*

|  |  | Original sample  (*N*=9206) | Attrition | | | | Final sample  (*N*=2349) |
| --- | --- | --- | --- | --- | --- | --- | --- |
|  |  |  | Responses only outside study period  (*N*=3566) | Baseline only responses  (*N*=1217) | Missing offspring’s gender information (*N*=36) | Survey completed only once (*N*=2038) |  |
| Location | | | |  |  |  |  |
|  | Greater London | 976 (10.6%) | 391 (11.0%) | 147 (12.1%) | 4 (11.1%) | 199 (9.8%) | 235 (10.0%) |
|  | Northern England | 1850 (20.1%) | 726 (20.4%) | 246 (20.2%) | 5 (13.9%) | 415 (20.4%) | 459 (19.5%) |
|  | Northern Ireland | 138 (1.5%) | 68 (1.9%) | 20 (1.6%) | 1 (2.8%) | 22 (1.1%) | 27 (1.1%) |
|  | Scotland | 581 (6.3%) | 228 (6.4%) | 92 (7.6%) | 3 (8.3%) | 135 (6.6%) | 123 (5.2%) |
|  | Southern England | 4185 (45.5%) | 1587 (44.5%) | 495 (40.7%) | 14 (38.9%) | 938 (46.0%) | 1150 (49.0%) |
|  | The Midlands | 1121 (12.2%) | 414 (11.6%) | 171 (14.1%) | 3 (8.3%) | 257 (12.6%) | 276 (11.7%) |
|  | Wales | 345 (3.7%) | 147 (4.1%) | 46 (3.8%) | 3 (8.3%) | 71 (3.5%) | 78 (3.3%) |
|  | Missing | 10 (0.1%) | 5 (0.0%) | 0 (0.0%) | 3 (8.3%) | 1 (0.0%) | 1 (0.0%) |
| Parent Gender | | | |  |  |  |  |
|  | Women | 8373 (90.9%) | 3157 (88.5%) | 1129 (92.8%) | 27 (75.0%) | 1894 (92.9%) | 2165 (92.2%) |
|  | Men | 764 (8.3%) | 377 (10.6%) | 82 (7.3%) | 3 (8.3%) | 130 (6.4%) | 173 (7.4%) |
|  | Other/Prefer not to say/Missing | 69 (0.7%) | 32 (0.9%) | 6 (0.5%) | 6 (16.7%) | 14 (0.6%) | 11 (0.4%) |
| Employment Status | | | |  |  |  |  |
|  | Self employed | 960 (10.4%) | 399 (11.2%) | 122 (10.0%) | 8 (22.2%) | 211 (10.4%) | 220 (9.4%) |
|  | Unemployed/Other | 1760 (19.1%) | 712 (20.0%) | 274 (22.5%) | 4 (11.1%) | 349 (17.1%) | 421 (17.9%) |
|  | Working full time | 3307 (35.9%) | 1351 (37.9%) | 438 (36.0%) | 10 (27.8%) | 737 (36.2%) | 771 (32.8%) |
|  | Working part time | 3169 (34.4%) | 1099 (30.8%) | 383 (31.5%) | 11 (30.6%) | 740 (36.3%) | 936 (39.8%) |
|  | Missing | 10 (0.1%) | 5 (0.1%) | 0 (0.0%) | 3 (8.3%) | 1 (0.0%) | 1 (0.0%) |
| Household Income | | | |  |  |  |  |
|  | <£16,000 | 987 (10.7%) | 416 (11.7%) | 197 (16.2%) | 6 (16.7%) | 182 (8.9%) | 186 (7.9%) |
|  | ≥£16,000 | 7593 (82.5%) | 2908 (81.5%) | 928 (76.3%) | 24 (66.7%) | 1736 (85.2%) | 1997 (85.0%) |
|  | Missing | 626 (6.8%) | 242 (6.8%) | 92 (7.6%) | 6 (16.7%) | 120 (5.9%) | 166 (7.1%) |
| Parent Ethnicity | | | |  |  |  |  |
|  | Ethnic minorities (excluding white minorities) | 683 (7.4%) | 336 (9.4%) | 133 (10.9%) | 2 (5.6%) | 107 (5.3%) | 104 (4.4%) |
|  | White | 8407 (91.3%) | 3180 (89.2%) | 1059 (87.0%) | 30 (83.3%) | 1914 (93.9%) | 2225 (94.7%) |
|  | Missing | 116 (1.3%) | 50 (1.4%) | 25 (2.1%) | 4 (11.1%) | 17 (0.8%) | 20 (0.9%) |
| Child Gender | | | |  |  |  |  |
|  | Girls | 4404 (47.8%) | 1700 (47.7%) | 584 (48.0%) | 0 (0.0%) | 978 (48.0%) | 1120 (47.7%) |
|  | Boys | 4733 (51.4%) | 1834 (51.4%) | 623 (51.2%) | 0 (0.0%) | 1060 (52.0%) | 1229 (52.3%) |
|  | Other/Prefer not to say/Missing | 69 (0.7%) | 32 (0.9%) | 10 (0.8%) | 36 (100.0%) | 0 (0.0%) | 0 (0.0%) |
| Child Age | | | |  |  |  |  |
|  | Primary school aged | 5837 (63.4%) | 2172 (60.9%) | 810 (66.6%) | 10 (27.8%) | 1204 (59.1%) | 1487 (63.3%) |
|  | Secondary school aged | 3368 (36.6%) | 1393 (39.1%) | 407 (33.4%) | 26 (72.2%) | 834 (40.9%) | 862 (36.7%) |
|  | Missing | 1 (0.0%) | 1 (0.0%) | 0 (0.0%) | 0 (0.0%) | (0.0%) | 1487 (63.3%) |
| SEN/ND Status | | | |  |  |  |  |
|  | No SEN/ND | 7433 (80.7%) | 2825 (79.2%) | 977 (80.3%) | 6 (16.7%) | 1543 (75.7%) | 1874 (79.8%) |
|  | SEN/ND | 1628 (17.7%) | 641 (18.0%) | 2018 (17.9%) | 12 (33.3%) | 350 (17.2%) | 366 (15.6%) |
|  | Missing | 145 (1.6%) | 100 (2.8%) | 22 (1.8%) | 18 (50.0%) | 145 (7.1%)* | 109 (4.6%) |
| Child Mental Health | | | |  |  |  |  |
|  | Depression/Anxiety/Other | 526 (5.7%) | 231 (6.5%) | 54 (4.4%) | 4 (11.1%) | 105 (5.2%) | 118 (5.0%) |
|  | No | 8523 (92.6%) | 3229 (90.5%) | 1139 (84.2%) | 14 (38.9%) | 1786 (87.6%) | 2120 (90.3%) |
|  | Missing | 157 (1.7%) | 106 (3.0%) | 24 (10.1%) | 18 (50.0%) | 147 (7.2%) | 111 (4.7%) |

*Note.* SEN/ND = Special Educational Needs/Neurodevelopmental Disorders. * Note, as some parents have reported on different children at the follow ups than the baseline, additional child-level demographic information may be missing at follow-up (in comparison to baseline) surveys.

**Figure S1**

*Graphic representations of the random intercept cross-lagged panel model (RI-CLPM) in the study*


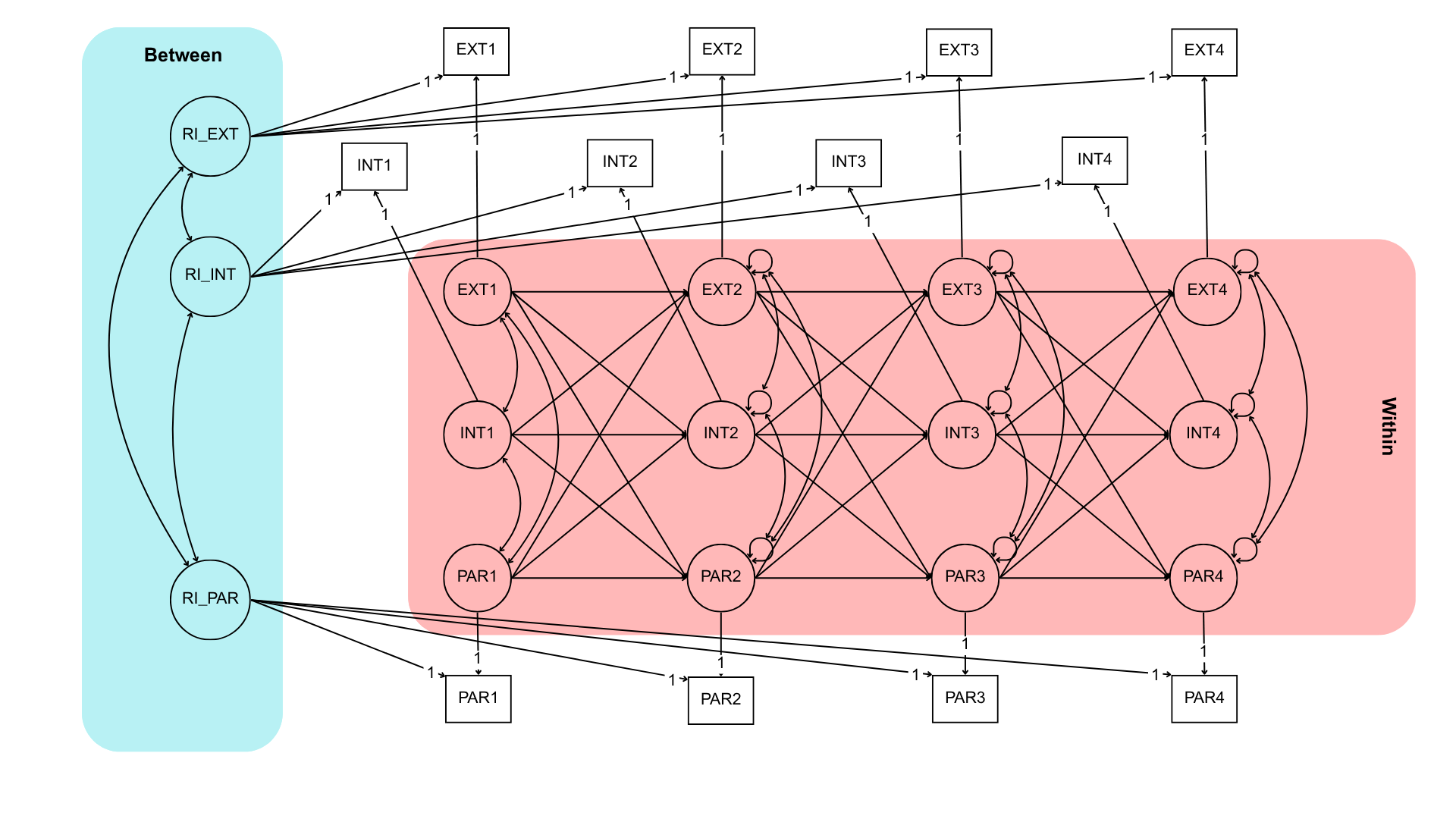


*Note.* Rectangles denote observed scores, whilst circles denote latent variables. INT = SDQ internalising symptoms. EXT = SDQ externalising symptoms. PAR = DASS score. RI = Random intercept. Numbers 1 to 4 refer to data collection time points (1 = May’20, 2 = Sep’20, 3 = Jan’21, 4 = May’21).

**Table S3**

*Cronbach’s alpha (α) for study questionnaires per study wave*

| Scale | Per wave | | | |  | Across waves | |
| --- | --- | --- | --- | --- | --- | --- | --- |
|  | W1 | W2 | W3 | W4 |  | *M* | *SD* |
| DASS-21 total score | .93 | .95 | .95 | .95 |  | .95 | .01 |
| SDQ internalising (emotional problem) symptoms | .81 | .83 | .81 | .84 |  | .82 | .02 |
| SDQ externalising symptoms | .83 | .85 | .84 | .85 |  | .84 | .01 |

## Model Selection

**Table S4**

*Model selection and fit indices for building Random Intercept Cross-Lagged Panel Model (RI-CLPM)*

| Model | Constraints added | *df* | AIC | BIC | CFI | TLI | RMSEA | SRMR | Δχ² | Δ*p* |
| --- | --- | --- | --- | --- | --- | --- | --- | --- | --- | --- |
| M0 | Base model | 69 | 108550 | 108948 | .997 | .991 | .028 | .018 | - | - |
| M1 | M0 + Residual errors constrained | 63 | 108624 | 108987 | .991 | .979 | .043 | .024 | 86.39 | <.001 |
| M2 | M0 + Residual covariances constrained | 63 | 108553 | 108916 | .997 | .992 | .027 | .020 | 14.70 | .023 |
| **M3** | **M0 + Cross-lagged constrained** | **57** | **108537** | **108865** | **.997** | **.995** | **.022** | **.021** | **11.01** | **.529** |
| M4 | M3 + Auto-regressions constrained | 51 | 108566 | 108860 | .995 | .991 | .028 | .029 | 40.81 | <.001 |

*Note.* M0 to M4 indicate the order of models for the χ² difference testing. **Bold** indicates acceptable model constraints.

**Table S5**

*Model selection and fit indices for multi-group random intercept cross-lagged panel model (RI-CLPM) for age*

| Model | Constraints added | *df* | AIC | BIC | CFI | TLI | RMSEA | SRMR | Δχ² | Δ*p* |
| --- | --- | --- | --- | --- | --- | --- | --- | --- | --- | --- |
| A1 | Base model (no constraints) | 138 | 108446 | 109241 | .996 | .988 | .032 | .022 | - | - |
| A2* | A1 + Cross-lagged constraints within and between groups | 108 | 108448 | 109070 | .994 | .989 | .031 | .028 | 61.93 | <.001 |
| A3 | A1 + Cross-lagged constraints within groups | 114 | 108445 | 109102 | .995 | .989 | .031 | .028 | 46.80 | .003 |
| A4 | A1 + Cross-lagged constraints between groups | 120 | 108460 | 109152 | .994 | .986 | .034 | .026 | 50.34 | <.001 |
| A5 | A1 + Means constraints between groups | 126 | 108523 | 109249 | .990 | .975 | .047 | .040 | 101.42 | <.001 |
| A6 | A1 + Cross-lagged constraints within secondary school aged group only | 126 | 108455 | 109181 | .995 | .987 | .034 | .026 | 33.21 | <.001 |
| **A7** | **A1 + Cross-lagged constraints within primary school aged group only** | **126** | **108436** | **109161** | **.996** | **.991** | **.029** | **.023** | **13.60** | **.327** |
| **A8** | **A7 + PAR on EXT constraints within secondary school aged group** | **124** | **108433** | **109147** | **.996** | **.991** | **.028** | **.023** | **1.04** | **.594** |
| A9 | A8 + INT on PAR constraints for  secondary school aged group | 122 | 108440 | 109143 | .996 | .990 | .030 | .026 | 11.09 | .004 |
| **A10** | **A8 + EXT on PAR constraints within secondary school aged group** | **122** | **108433** | **109136** | **.996** | **.991** | **.028** | **.024** | **4.95** | **.084** |
| A11 | A10 + PAR on INT constraints for  secondary school aged group | 120 | 108439 | 109130 | .995 | .990 | .029 | .026 | 9.07 | .011 |
| **A12** | **A10 + EXT on INT constraints for**  **secondary school aged group** | **120** | **108434** | **109125** | **.996** | **.991** | **.028** | **.024** | **4.19** | **.123** |
| A13 | A12 + INT on EXT constraints for  secondary school aged group | 118 | 108444 | 109124 | .995 | .989 | .031 | .025 | 14.28 | .001 |

*Note.* INT = SDQ internalising symptoms. EXT = SDQ externalising symptoms. PAR = DASS score. A1 to A12 indicate the order of models for the χ² difference testing. **Bold** indicates acceptable model constraints. * Maximum number of iterations were increased to 100,000 to enable convergence.

**Table S6**

*Model selection and fit indices for multi-group random intercept cross-lagged panel model (RI-CLPM) for gender*

| Model Constraints Added | | *df* | AIC | BIC | CFI | TLI | RMSEA | SRMR | Δχ² | Δ*p* |
| --- | --- | --- | --- | --- | --- | --- | --- | --- | --- | --- |
| G1 | Base model (no constraints) | 138 | 108500 | 109295 | .997 | .990 | .030 | .021 | - | - |
| **G2*** | **G1 + Cross-lagged constrained within and between groups** | **108** | **108477** | **109099** | **.996** | **.993** | **.024** | **.024** | **36.90** | **.180** |
| G3 | G2 + Means constrained between groups | 96 | 108564 | 109117 | .989 | .983 | .039 | .039 | 110.45 | <.001 |

*Note.* G1 to G3 indicate the order of models for the χ² difference testing. **Bold** indicates acceptable model constraints. * Maximum number of iterations were increased to 100,000 to enable convergence.

## Descriptive Statistics

**Table S7**

*Variances and covariances of study variables in the final sample based on a saturated model using full-information maximum likelihood (FIML) estimator in Mplus*

| Variable | 1 | 2 | 3 | 4 | 5 | 6 | 7 | 8 | 9 | 10 | 11 | 12 |
| --- | --- | --- | --- | --- | --- | --- | --- | --- | --- | --- | --- | --- |
| 1. INT1 | 6.83 |  |  |  |  |  |  |  |  |  |  |  |
| 2. INT2 | 5.05 | 6.83 |  |  |  |  |  |  |  |  |  |  |
| 3. INT3 | 4.87 | 5.11 | 6.74 |  |  |  |  |  |  |  |  |  |
| 4. INT4 | 4.82 | 5.32 | 5.31 | 7.26 |  |  |  |  |  |  |  |  |
| 5. EXT1 | 4.85 | 4.01 | 4.05 | 3.72 | 16.57 |  |  |  |  |  |  |  |
| 6. EXT2 | 4.33 | 4.88 | 4.28 | 4.37 | 13.53 | 16.18 |  |  |  |  |  |  |
| 7. EXT3 | 4.31 | 4.42 | 4.98 | 4.45 | 13.12 | 13.59 | 16.18 |  |  |  |  |  |
| 8. EXT4 | 4.39 | 4.62 | 4.48 | 5.26 | 12.60 | 13.51 | 13.76 | 16.47 |  |  |  |  |
| 9. PAR1 | 18.13 | 16.62 | 16.83 | 17.49 | 26.11 | 25.39 | 25.42 | 22.21 | 375.29 |  |  |  |
| 10. PAR2 | 17.48 | 21.70 | 20.32 | 21.24 | 25.02 | 29.65 | 28.03 | 26.29 | 304.37 | 448.96 |  |  |
| 11. PAR3 | 18.85 | 20.45 | 24.84 | 23.13 | 28.73 | 30.03 | 34.25 | 30.27 | 319.22 | 378.54 | 591.64 |  |
| 12. PAR4 | 18.35 | 20.40 | 21.12 | 25.43 | 24.46 | 28.31 | 28.14 | 31.55 | 299.34 | 365.36 | 408.35 | 534.17 |

*Note*. All variances and covariances were significant at *p* <.001. INT = SDQ internalising symptoms. EXT = SDQ externalising symptoms. PAR = DASS score. Numbers 1 to 4 refer to data collection time points (1 = May’20, 2 = Sep’20, 3 = Jan’21, 4 = May’21).

**Table S8**

*Variances and covariances of study variables by age based on a saturated model using full-information maximum likelihood (FIML) estimator in Mplus*

|  | Variable | 1 | 2 | 3 | 4 | 5 | 6 | 7 | 8 | 9 | 10 | 11 | 12 |
| --- | --- | --- | --- | --- | --- | --- | --- | --- | --- | --- | --- | --- | --- |
| Primary school aged | | | | |  |  |  |  |  |  |  |  |  |
|  | 1. INT1 | 6.26 |  |  |  |  |  |  |  |  |  |  |  |
|  | 2. INT2 | 4.50 | 6.28 |  |  |  |  |  |  |  |  |  |  |
|  | 3. INT3 | 4.34 | 4.55 | 6.20 |  |  |  |  |  |  |  |  |  |
|  | 4. INT4 | 4.08 | 4.70 | 4.64 | 6.73 |  |  |  |  |  |  |  |  |
|  | 5. EXT1 | 4.18 | 3.28 | 3.85 | 3.06 | 15.30 |  |  |  |  |  |  |  |
|  | 6. EXT2 | 3.85 | 4.43 | 4.08 | 3.90 | 12.54 | 16.16 |  |  |  |  |  |  |
|  | 7. EXT3 | 3.75 | 3.85 | 4.72 | 3.88 | 12.43 | 13.47 | 16.34 |  |  |  |  |  |
|  | 8. EXT4 | 3.81 | 4.10 | 4.25 | 4.68 | 11.84 | 13.38 | 13.71 | 16.32 |  |  |  |  |
|  | 9. PAR1 | 16.81 | 14.86 | 15.67 | 15.17 | 26.56 | 24.62 | 24.95 | 24.10 | 369.41 |  |  |  |
|  | 10. PAR2 | 15.50 | 18.68 | 17.00 | 17.89 | 23.43 | 27.55 | 25.09 | 25.89 | 287.53 | 421.80 |  |  |
|  | 11. PAR3 | 18.18 | 18.77 | 24.43 | 22.83 | 27.34 | 27.61 | 32.46 | 31.72 | 309.50 | 366.19 | 606.44 |  |
|  | 12. PAR4 | 15.35 | 16.00 | 17.36 | 21.69 | 24.34 | 27.33 | 25.11 | 32.37 | 291.54 | 334.32 | 392.82 | 511.37 |
| Secondary school aged | | | |  |  |  |  |  |  |  |  |  |  |
|  | 1. INT1 | 7.70 |  |  |  |  |  |  |  |  |  |  |  |
|  | 2. INT2 | 5.89 | 7.63 |  |  |  |  |  |  |  |  |  |  |
|  | 3. INT3 | 5.68 | 5.96 | 7.57 |  |  |  |  |  |  |  |  |  |
|  | 4. INT4 | 6.04 | 6.28 | 6.34 | 8.08 |  |  |  |  |  |  |  |  |
|  | 5. EXT1 | 5.74 | 5.29 | 4.16 | 4.72 | 17.34 |  |  |  |  |  |  |  |
|  | 6. EXT2 | 4.94 | 5.58 | 4.44 | 5.06 | 14.37 | 15.65 |  |  |  |  |  |  |
|  | 7. EXT3 | 5.02 | 5.38 | 5.23 | 5.25 | 13.33 | 13.18 | 15.23 |  |  |  |  |  |
|  | 8. EXT4 | 5.27 | 5.49 | 4.75 | 6.16 | 13.05 | 13.18 | 13.25 | 16.24 |  |  |  |  |
|  | 9. PAR1 | 19.48 | 19.43 | 17.78 | 20.73 | 22.25 | 24.78 | 23.27 | 16.75 | 376.95 |  |  |  |
|  | 10. PAR2 | 19.94 | 26.28 | 25.69 | 26.57 | 27.59 | 32.58 | 32.27 | 27.35 | 330.30 | 492.90 |  |  |
|  | 11. PAR3 | 19.57 | 23.01 | 25.19 | 23.06 | 27.02 | 31.84 | 34.58 | 26.05 | 325.05 | 393.62 | 562.61 |  |
|  | 12. PAR4 | 23.69 | 27.44 | 26.83 | 31.24 | 25.38 | 28.85 | 32.47 | 30.40 | 302.83 | 411.63 | 428.02 | 562.34 |

*Note*. All variances and covariances were significant at *p* <.001. INT = SDQ internalising symptoms. EXT = SDQ externalising symptoms. PAR = DASS score. Numbers 1 to 4 refer to data collection time points (1 = May’20, 2 = Sep’20, 3 = Jan’21, 4 = May’21).

**Table S9**

*Variances and covariances of study variables by gender based on a saturated model using full-information maximum likelihood (FIML) estimator in Mplus*

|  | Variable | 1 | 2 | 3 | 4 | 5 | 6 | 7 | 8 | 9 | 10 | 11 | 12 |
| --- | --- | --- | --- | --- | --- | --- | --- | --- | --- | --- | --- | --- | --- |
| Boys | |  |  |  |  |  |  |  |  |  |  |  |  |
|  | 1. INT1 | 6.51 |  |  |  |  |  |  |  |  |  |  |  |
|  | 2. INT2 | 4.67 | 6.35 |  |  |  |  |  |  |  |  |  |  |
|  | 3. INT3 | 4.69 | 4.88 | 6.50 |  |  |  |  |  |  |  |  |  |
|  | 4. INT4 | 4.51 | 4.98 | 4.93 | 6.85 |  |  |  |  |  |  |  |  |
|  | 5. EXT1 | 4.64 | 3.88 | 4.23 | 3.86 | 16.86 |  |  |  |  |  |  |  |
|  | 6. EXT2 | 3.98 | 4.71 | 4.36 | 4.15 | 13.73 | 16.41 |  |  |  |  |  |  |
|  | 7. EXT3 | 4.22 | 4.13 | 5.06 | 4.23 | 12.94 | 13.42 | 15.92 |  |  |  |  |  |
|  | 8. EXT4 | 4.21 | 4.40 | 4.54 | 5.16 | 12.95 | 13.43 | 13.53 | 16.25 |  |  |  |  |
|  | 9. PAR1 | 18.25 | 16.81 | 17.65 | 17.00 | 25.22 | 25.45 | 24.27 | 23.08 | 355.19 |  |  |  |
|  | 10. PAR2 | 17.92 | 21.45 | 21.49 | 20.02 | 24.06 | 29.13 | 27.24 | 25.26 | 294.23 | 418.19 |  |  |
|  | 11. PAR3 | 19.82 | 20.59 | 27.21 | 25.15 | 24.44 | 28.02 | 33.08 | 30.57 | 311.28 | 358.36 | 596.64 |  |
|  | 12. PAR4 | 15.71 | 19.24 | 21.49 | 24.02 | 23.39 | 27.29 | 27.85 | 31.14 | 289.14 | 338.27 | 410.86 | 521.39 |
| Girls | |  |  |  |  |  |  |  |  |  |  |  |  |
|  | 1. INT1 | 7.17 |  |  |  |  |  |  |  |  |  |  |  |
|  | 2. INT2 | 5.43 | 7.30 |  |  |  |  |  |  |  |  |  |  |
|  | 3. INT3 | 4.98 | 5.31 | 6.91 |  |  |  |  |  |  |  |  |  |
|  | 4. INT4 | 5.09 | 5.59 | 5.62 | 7.58 |  |  |  |  |  |  |  |  |
|  | 5. EXT1 | 5.30 | 4.27 | 4.10 | 3.81 | 15.61 |  |  |  |  |  |  |  |
|  | 6. EXT2 | 4.90 | 5.14 | 4.41 | 4.78 | 12.67 | 15.28 |  |  |  |  |  |  |
|  | 7. EXT3 | 4.67 | 4.87 | 5.15 | 4.95 | 12.77 | 13.20 | 15.97 |  |  |  |  |  |
|  | 8. EXT4 | 4.78 | 4.92 | 4.62 | 5.55 | 11.66 | 12.98 | 13.49 | 16.17 |  |  |  |  |
|  | 9. PAR1 | 18.12 | 16.35 | 15.91 | 17.84 | 27.23 | 25.65 | 27.17 | 21.43 | 398.16 |  |  |  |
|  | 10. PAR2 | 17.13 | 21.72 | 18.88 | 21.98 | 25.63 | 29.99 | 28.85 | 26.68 | 315.24 | 479.51 |  |  |
|  | 11. PAR3 | 18.32 | 20.35 | 22.62 | 21.41 | 33.12 | 31.64 | 35.49 | 29.59 | 331.60 | 395.21 | 587.95 |  |
|  | 12. PAR4 | 20.83 | 21.33 | 20.59 | 26.75 | 24.85 | 28.76 | 28.38 | 31.49 | 308.78 | 392.30 | 407.57 | 546.32 |

*Note*. All variances and covariances were significant at *p* <.001. INT = SDQ internalising symptoms. EXT = SDQ externalising symptoms. PAR = DASS score. Numbers 1 to 4 refer to data collection time points (1 = May’20, 2 = Sep’20, 3 = Jan’21, 4 = May’21).

**Table S10**

*Means and standard deviations per wave and by offspring gender and age*

| Variable | Total sample | | |  | Primary | | |  | Secondary | | |  | Boys | | |  | Girls | | |
| --- | --- | --- | --- | --- | --- | --- | --- | --- | --- | --- | --- | --- | --- | --- | --- | --- | --- | --- | --- |
|  | *n* | *M* | *SD* |  | *n* | *M* | *SD* |  | *n* | *M* | *SD* |  | *n* | *M* | *SD* |  | *n* | *M* | *SD* |
| INT1 | 1378 | 2.98 | 2.62 |  | 876 | 3.08 | 2.52 |  | 502 | 2.81 | 2.78 |  | 726 | 2.84* | 2.54 |  | 652 | 3.13* | 2.70 |
| INT2 | 1745 | 2.72 | 2.64 |  | 1073 | 2.68 | 2.53 |  | 672 | 2.79 | 2.80 |  | 905 | 2.59 | 2.53 |  | 840 | 2.87 | 2.74 |
| INT3 | 1742 | 2.92 | 2.57 |  | 1036 | 3.01 | 2.46 |  | 706 | 2.78 | 2.72 |  | 910 | 2.71* | 2.52 |  | 832 | 3.15* | 2.61 |
| INT4 | 1459 | 2.79 | 2.66 |  | 900 | 2.83 | 2.55 |  | 559 | 2.72 | 2.84 |  | 763 | 2.60* | 2.59 |  | 696 | 3.00* | 2.73 |
| EXT1 | 1378 | 6.68 | 4.08 |  | 876 | 7.25** | 3.96 |  | 502 | 5.67** | 4.11 |  | 726 | 7.19 | 4.15 |  | 652 | 6.10 | 3.92 |
| EXT2 | 1745 | 5.90 | 4.06 |  | 1073 | 6.22** | 4.09 |  | 672 | 5.38** | 3.95 |  | 905 | 6.34 | 4.09 |  | 840 | 5.41 | 3.97 |
| EXT3 | 1742 | 6.25 | 4.03 |  | 1036 | 6.75** | 4.02 |  | 706 | 5.52** | 3.92 |  | 910 | 6.67 | 3.97 |  | 832 | 5.79 | 4.03 |
| EXT4 | 1459 | 5.75 | 4.02 |  | 900 | 6.15** | 3.99 |  | 559 | 5.10** | 3.99 |  | 763 | 6.19 | 4.03 |  | 696 | 5.26 | 3.96 |
| PAR1 | 1349 | 25.45 | 19.01 |  | 852 | 26.55 | 18.92 |  | 497 | 23.55 | 19.03 |  | 703 | 24.88* | 18.21 |  | 646 | 26.06* | 19.84 |
| PAR2 | 1706 | 23.06 | 21.19 |  | 1050 | 23.25 | 20.47 |  | 656 | 22.74 | 22.30 |  | 886 | 22.77 | 20.29 |  | 820 | 23.36 | 22.12 |
| PAR3 | 1721 | 30.12 | 24.45 |  | 1023 | 31.58* | 24.74 |  | 698 | 27.98* | 23.87 |  | 901 | 30.05 | 24.56 |  | 820 | 30.20 | 24.34 |
| PAR4 | 1444 | 25.64 | 23.07 |  | 890 | 25.67 | 22.38 |  | 554 | 25.60 | 24.15 |  | 756 | 25.74 | 22.88 |  | 688 | 25.55 | 23.28 |

** p ≤ .05 ** p ≤ .01. Note*. Primary = Primary school aged children (4-10 years old). Secondary = Secondary school aged children (11-16 years old). INT = SDQ internalising symptoms. EXT = SDQ externalising symptoms. PAR = DASS score. Numbers 1 to 4 refer to data collection time points (1 = May’20, 2 = Sep’20, 3 = Jan’21, 4 = May’21).

## Estimated Pathways

**Table S11**

*Auto-regressive, concurrent, and cross-lagged paths, as well as variances and residual variances, in the main random intercept cross-lagged panel model (RI-CLPM)*

| Parameter | | | Estimate | *SE* | *p* | β |
| --- | --- | --- | --- | --- | --- | --- |
| Between-individual effects | | |  |  |  |  |
|  | Covariances between random intercepts | |  |  |  |  |
|  |  | RI_INT – RI_EXT | 4.32 | 0.22 | **<.001** | .527 |
|  |  | RI_INT – RI_PAR | 18.54 | 1.11 | **<.001** | .454 |
|  |  | RI_PAR– RI_EXT | 26.02 | 1.72 | **<.001** | .393 |
|  | Variances of random intercepts | |  |  |  |  |
|  |  | RI_INT | 5.07 | 0.18 | **<.001** |  |
|  |  | RI_EXT | 13.27 | 0.45 | **<.001** |  |
|  |  | RI_PAR | 329.55 | 12.92 | **<.001** |  |
| Within-individual effects | | |  |  |  |  |
|  | Concurrent paths (covariances) | |  |  |  |  |
|  |  | INT 1 – EXT 1 | 0.91 | 0.12 | **<.001** | .309 |
|  |  | INT 2 – EXT 2 | 0.46 | 0.10 | **<.001** | .227 |
|  |  | INT 3 – EXT 3 | 0.57 | 0.11 | **<.001** | .275 |
|  |  | INT 4 – EXT 4 | 0.81 | 0.10 | **<.001** | .319 |
|  |  | INT 1 – PAR 1 | 2.13 | 0.62 | **<.001** | .173 |
|  |  | INT 2 – PAR 2 | 2.61 | 0.73 | **<.001** | .210 |
|  |  | INT 3 – PAR 3 | 4.61 | 0.77 | **<.001** | .233 |
|  |  | INT 4 – PAR 4 | 4.15 | 0.67 | **<.001** | .218 |
|  |  | PAR 1 – EXT 1 | 2.50 | 0.88 | **.004** | .148 |
|  |  | PAR 2 – EXT 2 | 2.75 | 0.94 | **.003** | .173 |
|  |  | PAR 3 – EXT 3 | 4.70 | 1.01 | **<.001** | .188 |
|  |  | PAR 4 – EXT 4 | 5.18 | 0.86 | **<.001** | .219 |
|  | Auto-regressive paths | |  |  |  |  |
|  |  | INT 1 – INT 2 | 0.04 | 0.05 | .419 | .047 |
|  |  | INT 2 – INT 3 | -0.04 | 0.07 | .552 | -.038 |
|  |  | INT 3 – INT 4 | 0.10 | 0.06 | .092 | .089 |
|  |  | EXT 1 – EXT 2 | 0.14 | 0.05 | **.007** | .169 |
|  |  | EXT 2 – EXT 3 | 0.03 | 0.08 | .711 | .029 |
|  |  | EXT 3 – EXT 4 | 0.13 | 0.07 | .070 | .114 |
|  |  | PAR 1 – PAR 2 | -0.28 | 0.17 | .094 | -.229 |
|  |  | PAR 2 – PAR 3 | 0.36 | 0.08 | **<.001** | .229 |
|  |  | PAR 3 – PAR 4 | 0.28 | 0.03 | **<.001** | .318 |
|  | Cross-lagged paths | | | | | |
|  |  | INT 1 – EXT 2 | -0.05 | 0.04 | .199 | -.045 |
|  |  | INT 2 – EXT 3 | -0.05 | 0.04 | .199 | -.039 |
|  |  | INT 3 – EXT 4 | -0.05 | 0.04 | .199 | -.036 |
|  |  | EXT 1 – INT 2 | -0.02 | 0.02 | .352 | -.036 |
|  |  | EXT 2 – INT 3 | -0.02 | 0.02 | .352 | -.028 |
|  |  | EXT 3 – INT 4 | -0.02 | 0.02 | .352 | -.025 |
|  |  | INT 1 – PAR 2 | -0.12 | 0.30 | .695 | -.017 |
|  |  | INT 2 – PAR 3 | -0.12 | 0.30 | .695 | -.009 |
|  |  | INT 3 – PAR 4 | -0.12 | 0.30 | .695 | -.011 |
|  |  | PAR 1 – INT 2 | 0.01 | <0.01 | **.034** | .049 |
|  |  | PAR 2 – INT 3 | 0.01 | <0.01 | **.034** | .058 |
|  |  | PAR 3 – INT 4 | 0.01 | <0.01 | **.034** | .081 |
|  |  | EXT 1 – PAR 2 | -0.04 | 0.25 | .881 | -.007 |
|  |  | EXT 2 – PAR 3 | -0.04 | 0.25 | .881 | -.004 |
|  |  | EXT 3 – PAR 4 | -0.04 | 0.25 | .881 | -.004 |
|  |  | PAR 1 – EXT 2 | 0.01 | 0.01 | .068 | .043 |
|  |  | PAR 2 – EXT 3 | 0.01 | 0.01 | .068 | .052 |
|  |  | PAR 3 – EXT 4 | 0.01 | 0.01 | .068 | .073 |
|  | Variances | |  |  |  |  |
|  |  | INT 1 | 2.12 | 0.14 | **<.001** |  |
|  |  | EXT 1 | 4.05 | 0.27 | **<.001** |  |
|  |  | PAR 1 | 70.90 | 10.26 | **<.001** |  |
|  | Residual variances | |  |  |  |  |
|  |  | INT 2 | 1.58 | 0.12 | **<.001** |  |
|  |  | INT 3 | 1.66 | 0.14 | **<.001** |  |
|  |  | INT 4 | 2.03 | 0.11 | **<.001** |  |
|  |  | EXT 2 | 2.60 | 0.22 | **<.001** |  |
|  |  | EXT 3 | 2.63 | 0.27 | **<.001** |  |
|  |  | EXT 4 | 3.14 | 0.21 | **<.001** |  |
|  |  | PAR 2 | 97.39 | 15.99 | **<.001** |  |
|  |  | PAR 3 | 237.74 | 10.39 | **<.001** |  |
|  |  | PAR 4 | 178.85 | 8.81 | **<.001** |  |

*Note.* INT = SDQ internalising symptoms. EXT = SDQ externalising symptoms. PAR = DASS score. RI = Random intercept. Numbers 1 to 4 refer to data collection time points (1 = May’20, 2 = Sep’20, 3 = Jan’21, 4 = May’21). **Bold** indicates significant pathways.

**Table S12**

*Auto-regressive, concurrent, and cross-lagged paths, as well as variances and residual variances, in the age-moderated random intercept cross-lagged panel model (RI-CLPM)*

|  | | | Primary school aged (4-10 years old) | | | |  | Secondary school aged (11-16 years old) | | | |
| --- | --- | --- | --- | --- | --- | --- | --- | --- | --- | --- | --- |
| Parameter | | | Estimate | *SE* | *p* | β |  | Estimate | *SE* | *p* | β |
| Between-individual effects | | |  |  |  |  |  |  |  |  |  |
|  | Covariances between random intercepts | |  |  |  |  |  |  |  |  |  |
|  |  | RI_INT – RI_EXT | 3.83 | 0.26 | **<.001** | .510 |  | 5.14 | 0.38 | **<.001** | .576 |
|  |  | RI_INT – RI_PAR | 15.47 | 1.31 | **<.001** | .419 |  | 23.43 | 2.04 | **<.001** | .514 |
|  |  | RI_PAR– RI_EXT | 25.20 | 2.12 | **<.001** | .403 |  | 24.56 | 3.01 | **<.001** | .367 |
|  | Variances of random intercepts | |  |  |  |  |  |  |  |  |  |
|  |  | RI_INT | 4.44 | 0.21 | **<.001** |  |  | 6.07 | 0.32 | **<.001** |  |
|  |  | RI_EXT | 12.73 | 0.56 | **<.001** |  |  | 13.09 | 0.70 | **<.001** |  |
|  |  | RI_PAR | 307.66 | 15.96 | **<.001** |  |  | 341.90 | 24.38 | **<.001** |  |
| Within-individual effects | | |  |  |  |  |  |  |  |  |  |
|  | Concurrent paths (covariances) | |  |  |  |  |  |  |  |  |  |
|  |  | INT 1 – EXT 1 | 0.78 | 0.15 | **<.001** | .277 |  | 1.05 | 0.22 | **<.001** | .366 |
|  |  | INT 2 – EXT 2 | 0.50 | 0.14 | **<.001** | .231 |  | 0.27 | 0.18 | .143 | .168 |
|  |  | INT 3 – EXT 3 | 0.63 | 0.14 | **<.001** | .271 |  | 0.05 | 0.19 | .816 | .028 |
|  |  | INT 4 – EXT 4 | 0.76 | 0.13 | **<.001** | .280 |  | 0.98 | 0.19 | **<.001** | .429 |
|  |  | INT 1 – PAR 1 | 1.90 | 0.81 | **.020** | .151 |  | -1.38 | 1.67 | .410 | -.131 |
|  |  | INT 2 – PAR 2 | 3.52 | 0.92 | **<.001** | .268 |  | -1.79 | 2.83 | .526 | -.172 |
|  |  | INT 3 – PAR 3 | 6.18 | 1.02 | **<.001** | .299 |  | 4.72 | 1.45 | **.001** | .256 |
|  |  | INT 4 – PAR 4 | 4.20 | 0.88 | **<.001** | .211 |  | 5.37 | 1.11 | **<.001** | .306 |
|  |  | PAR 1 – EXT 1 | 2.31 | 1.10 | **.036** | .141 |  | 2.41 | 1.79 | .179 | .145 |
|  |  | PAR 2 – EXT 2 | 2.26 | 1.28 | .076 | .129 |  | 3.99 | 1.59 | **.012** | .266 |
|  |  | PAR 3 – EXT 3 | 4.88 | 1.35 | **<.001** | .167 |  | 7.45 | 1.66 | **<.001** | .420 |
|  |  | PAR 4 – EXT 4 | 5.76 | 1.10 | **<.001** | .239 |  | 5.54 | 1.49 | **<.001** | .247 |
|  | Auto-regressive paths | |  |  |  |  |  |  |  |  |  |
|  |  | INT 1 – INT 2 | 0.06 | 0.06 | .326 | .068 |  | -0.22 | 0.18 | .243 | -.249 |
|  |  | INT 2 – INT 3 | -0.04 | 0.08 | .650 | -.037 |  | -<0.01 | 0.11 | .982 | -.002 |
|  |  | INT 3 – INT 4 | 0.08 | 0.08 | .334 | .064 |  | 0.20 | 0.07 | **.006** | .200 |
|  |  | EXT 1 – EXT 2 | 0.05 | 0.08 | .559 | .050 |  | 0.39 | 0.06 | **<.001** | .481 |
|  |  | EXT 2 – EXT 3 | 0.13 | 0.09 | .180 | .117 |  | -0.01 | 0.11 | .896 | -.018 |
|  |  | EXT 3 – EXT 4 | 0.23 | 0.07 | **.001** | .227 |  | -0.22 | 0.20 | .265 | -.163 |
|  |  | PAR 1 – PAR 2 | -0.20 | 0.19 | .306 | -.162 |  | -0.37 | 0.46 | .428 | -.256 |
|  |  | PAR 2 – PAR 3 | 0.47 | 0.09 | **<.001** | .292 |  | 0.33 | 0.11 | **.002** | .242 |
|  |  | PAR 3 – PAR 4 | 0.26 | 0.04 | **<.001** | .313 |  | 0.31 | 0.06 | **<.001** | .326 |
|  | Cross-lagged paths | | | | | |  |  |  |  |  |
|  |  | INT 1 – EXT 2 | -0.06 | 0.05 | .288 | -.048 |  | -0.17 | 0.07 | **.024** | -.129 |
|  |  | INT 2 – EXT 3 | -0.06 | 0.05 | .288 | -.039 |  | -0.17 | 0.07 | **.024** | -.149 |
|  |  | INT 3 – EXT 4 | -0.06 | 0.05 | .288 | -.039 |  | -0.17 | 0.07 | **.024** | -.126 |
|  |  | EXT 1 – INT 2 | -0.04 | 0.03 | .228 | -.054 |  | 0.13 | 0.07 | .060 | .244 |
|  |  | EXT 2 – INT 3 | -0.04 | 0.03 | .228 | -.048 |  | -0.20 | 0.09 | **.024** | -.257 |
|  |  | EXT 3 – INT 4 | -0.04 | 0.03 | .228 | -.044 |  | -0.07 | 0.11 | .520 | -.065 |
|  |  | INT 1 – PAR 2 | 0.18 | 0.37 | .623 | .025 |  | -3.61 | 1.62 | **.025** | -.437 |
|  |  | INT 2 – PAR 3 | 0.18 | 0.37 | .623 | .014 |  | -0.62 | 0.98 | .529 | -.047 |
|  |  | INT 3 – PAR 4 | 0.18 | 0.37 | .623 | .017 |  | 0.97 | 0.76 | .202 | .089 |
|  |  | PAR 1 – INT 2 | 0.01 | <0.01 | **.002** | .088 |  | -0.06 | 0.04 | .134 | -.413 |
|  |  | PAR 2 – INT 3 | 0.01 | <0.01 | **.002** | .107 |  | 0.03 | 0.01 | **.028** | .231 |
|  |  | PAR 3 – INT 4 | 0.01 | <0.01 | **.002** | .147 |  | <0.01 | 0.01 | .732 | .029 |
|  |  | EXT 1 – PAR 2 | -0.48 | 0.28 | .090 | -.087 |  | 1.67 | 0.55 | **.002** | .320 |
|  |  | EXT 2 – PAR 3 | -0.48 | 0.28 | .090 | -.048 |  | 1.67 | 0.55 | **.002** | .189 |
|  |  | EXT 3 – PAR 4 | -0.48 | 0.28 | .090 | -.062 |  | 1.67 | 0.55 | **.002** | .148 |
|  |  | PAR 1 – EXT 2 | 0.01 | 0.01 | .153 | .039 |  | 0.03 | 0.01 | **.005** | .133 |
|  |  | PAR 2 – EXT 3 | 0.01 | 0.01 | .153 | .044 |  | 0.03 | 0.01 | **.005** | .256 |
|  |  | PAR 3 – EXT 4 | 0.01 | 0.01 | .153 | .069 |  | 0.03 | 0.01 | **.005** | .258 |
|  | Variances | |  |  |  |  |  |  |  |  |  |
|  |  | INT 1 | 2.15 | 0.17 | **<.001** |  |  | 1.82 | 0.25 | **<.001** |  |
|  |  | EXT 1 | 3.66 | 0.35 | **<.001** |  |  | 4.55 | 0.44 | **<.001** |  |
|  |  | PAR 1 | 73.51 | 13.05 | **<.001** |  |  | 60.61 | 20.37 | **.003** |  |
|  | Residual variances | |  |  |  |  |  |  |  |  |  |
|  |  | INT 2 | 1.63 | 0.16 | **<.001** |  |  | 1.10 | 0.35 | **.002** |  |
|  |  | INT 3 | 1.65 | 0.17 | **<.001** |  |  | 1.65 | 0.23 | **<.001** |  |
|  |  | INT 4 | 2.24 | 0.16 | **<.001** |  |  | 1.79 | 0.16 | **<.001** |  |
|  |  | EXT 2 | 2.88 | 0.33 | **<.001** |  |  | 2.27 | 0.24 | **<.001** |  |
|  |  | EXT 3 | 3.30 | 0.32 | **<.001** |  |  | 1.52 | 0.39 | **<.001** |  |
|  |  | EXT 4 | 3.26 | 0.25 | **<.001** |  |  | 2.92 | 0.43 | **<.001** |  |
|  |  | PAR 2 | 106.10 | 18.82 | **<.001** |  |  | 98.93 | 36.98 | **.007** |  |
|  |  | PAR 3 | 259.47 | 13.84 | **<.001** |  |  | 206.02 | 14.88 | **<.001** |  |
|  |  | PAR 4 | 178.15 | 11.23 | **<.001** |  |  | 172.46 | 13.48 | **<.001** |  |
|  | Indirect effects * | |  |  |  |  |  |  |  |  |  |
|  |  | PAR 1 – EXT 2 – PAR 3 | -<0.01 | <0.01 | .350 | -.002 |  | 0.05 | 0.04 | .253 | .025 |
|  |  | PAR 2 – EXT 3 – PAR 4 | -<0.01 | <0.01 | .350 | -.003 |  | 0.05 | 0.04 | .253 | .038 |
|  |  | EXT 1 – PAR 2 – EXT 3 | -<0.01 | <0.01 | .350 | -.004 |  | 0.05 | 0.04 | .253 | .082 |
|  |  | EXT 2 – PAR 3 – EXT 4 | -<0.01 | <0.01 | .350 | -.003 |  | 0.05 | 0.04 | .253 | .049 |

*Note.* INT = SDQ internalising symptoms. EXT = SDQ externalising symptoms. PAR = DASS score. RI = Random intercept. Numbers 1 to 4 refer to data collection time points (1 = May’20, 2 = Sep’20, 3 = Jan’21, 4 = May’21). **Bold** indicates significant pathways. * Indirect effects were assessed using bootstrapping method with 5,000 samples.

## Appendix S1

## Sensitivity Analysis: Autoregressive Cross-Lagged Panel Model (CLPM)

### Model Selection Process and Summary for CLPM

Sensitivity analyses were performed using a traditional, autoregressive cross-lagged panel model (CLPM) as an alternative approach to the RI-CLPM models reported in the manuscript. We employed four-wave CLPM with additional two- and three-wave stability paths to examine the relationships between parent and offspring mental health symptoms, including all three study variables (parent internalising symptoms, offspring internalising symptoms, and offspring externalising symptoms). Otherwise, our approach to CLPM was similar to that of RI-CLPM reported in the main manuscript and conducted in line with the analysis plans outlined in the pre-registration: <https://doi.org/10.17605/OSF.IO/AJEXN>.

Full details of model indices for model selection can be found in Table S2 in Supporting Information. Equality constraints were imposed on cross-lagged paths to evaluate whether these relations were time-invariant. Imposing such constraints did not lead to a significantly worse model fit (Δ*χ^2^*(12) *=* 12.70, *p* = .391). Hence, all the cross-lagged paths were constrained to be equal over time in the final model, which showed good model fit.

#### Direct effects in the main CLPM

Higher offspring internalising, but not offspring externalising symptoms, predicted significantly higher subsequent parent internalising symptoms in subsequent waves, with small effect sizes (β: .030 - .035). Yet, higher parent internalising symptoms significantly predicted both, higher subsequent offspring internalising (medium effects; β: .055 - .067) and offspring externalising (small effects; β: .021 - .025) symptom scores. There were also significant bidirectional effects between offspring internalising and externalising symptoms. Yet, due to our focus on transactional parent-offspring interchange in this paper and the sake of brevity, we will omit interpretation of these pathways. See Table S13 for other pathways and a full list of standardised and unstandardized coefficients.

### Moderation by Age

#### Model Selection

We examined whether associations between parent internalising symptoms, offspring internalising symptoms and offspring externalising symptoms differed according to offspring age. Full model paths by age group are presented in Table S15. Similarly to RI-CLPM, we found that they varied between primary school aged (4-10 years) and secondary school aged offspring (11-16 years), Δχ *^2^*(30) = 46.39, *p* = .029 (Tables S14). However, what pathways were significant differed from the RI-CLPM as outlined below.

**Table S13**

*Model selection and fit indices for building Cross-Lagged Panel Model (CLPM)*

| Model | Pathway/Constraints added | *df* | AIC | BIC | CFI | TLI | RMSEA | SRMR | Δχ² | Δ*p* |
| --- | --- | --- | --- | --- | --- | --- | --- | --- | --- | --- |
| CLPM0 | Base model | 72 | 108518 | 108933 | 1.00 | .999 | .008 | .006 | - | - |
| **CLPM1** | **CLPM0 + Cross-lagged constrained** | **60** | **108507** | **108852** | **1.00** | **.999** | **.007** | **.010** | **12.70** | **.391** |
| CLPM2 | CLPM1 + Auto-regressions constrained | 54 | 108885 | 109196 | .970 | .947 | .068 | .047 | 390.06 | <.001 |
| CLPM3 | CLPM1 + Co-occurrent constrained | 51 | 108814 | 109108 | .975 | .960 | .059 | .112 | 324.90 | <.001 |

*Note.* CLPM0 to CLPM3 indicate the order of models for the χ² difference testing. **Bold** indicates acceptable model constraints.

**Table S14**

*Auto-regressive, concurrent, and cross-lagged paths in the main CLPM model*

| Parameters | | | *B* | *SE* | *p* | β |
| --- | --- | --- | --- | --- | --- | --- |
| Concurrent paths | | |  |  |  |  |
|  | | INT 1 – EXT 1 | 4.90 | 0.29 | **<.001** | .460 |
|  | | INT 2 – EXT 2 | 1.31 | 0.12 | **<.001** | .331 |
|  | | INT 3 – EXT 3 | 0.98 | 0.09 | **<.001** | .304 |
|  | | INT 4 – EXT 4 | 0.98 | 0.10 | **<.001** | .316 |
|  | | INT 1 – PAR 1 | 18.44 | 1.36 | **<.001** | .363 |
|  | | INT 2 – PAR 2 | 6.22 | 0.75 | **<.001** | .252 |
|  | | INT 3 – PAR 3 | 5.37 | 0.72 | **<.001** | .215 |
|  | | INT 4 – PAR 4 | 4.53 | 0.65 | **<.001** | .208 |
|  | | PAR 1 – EXT 1 | 26.81 | 2.08 | **<.001** | .339 |
|  | | PAR 2 – EXT 2 | 6.03 | 0.99 | **<.001** | .189 |
|  | | PAR 3 – EXT 3 | 5.48 | 0.93 | **<.001** | .170 |
|  | | PAR 4 – EXT 4 | 5.52 | 0.83 | **<.001** | .199 |
| Auto-regressive paths | | |  |  |  |  |
|  | Single-wave | |  |  |  |  |
|  | | INT 1 – INT 2 | 0.70 | 0.02 | **<.001** | .705 |
|  | | INT 2 – INT 3 | 0.46 | 0.03 | **<.001** | .464 |
|  | | INT 3 – INT 4 | 0.40 | 0.03 | **<.001** | .380 |
|  | | EXT 1 – EXT 2 | 0.79 | 0.02 | **<.001** | .804 |
|  | | EXT 2 – EXT 3 | 0.55 | 0.03 | **<.001** | .546 |
|  | | EXT 3 – EXT 4 | 0.45 | 0.03 | **<.001** | .447 |
|  | | PAR 1 – PAR 2 | 0.78 | 0.02 | **<.001** | .720 |
|  | | PAR 2 – PAR 3 | 0.57 | 0.04 | **<.001** | .499 |
|  | | PAR 3 – PAR 4 | 0.32 | 0.03 | **<.001** | .339 |
|  | Two-wave | |  |  |  |  |
|  | | INT 1 – INT 3 | 0.34 | 0.03 | **<.001** | .343 |
|  | | INT 2– INT 4 | 0.34 | 0.03 | **<.001** | .332 |
|  | | EXT 1 – EXT 3 | 0.32 | 0.03 | **<.001** | .329 |
|  | | EXT 2 – EXT 4 | 0.33 | 0.03 | **<.001** | .326 |
|  | | PAR 1 – PAR 3 | 0.36 | 0.05 | **<.001** | .289 |
|  | | PAR 2 – PAR 4 | 0.40 | 0.04 | **<.001** | .362 |
|  | Three-wave | |  |  |  |  |
|  | | INT 1 – INT 4 | 0.14 | 0.03 | **<.001** | .132 |
|  | | EXT 1 – EXT 4 | 0.12 | 0.03 | **<.001** | .123 |
|  | | PAR 1 – PAR 4 | 0.19 | 0.05 | **<.001** | .156 |
| Cross-lagged paths | | | | | | |
|  | | INT 1 – EXT 2 | 0.05 | 0.02 | **.001** | .033 |
|  | | INT 2 – EXT 3 | 0.05 | 0.02 | **.001** | .033 |
|  | | INT 3 – EXT 4 | 0.05 | 0.02 | **.001** | .032 |
|  | | EXT 1 – INT 2 | 0.02 | 0.01 | **.018** | .028 |
|  | | EXT 2 – INT 3 | 0.02 | 0.01 | **.018** | .027 |
|  | | EXT 3 – INT 4 | 0.02 | 0.01 | **.018** | .026 |
|  | | INT 1 – PAR 2 | 0.28 | 0.11 | **.010** | .035 |
|  | | INT 2 – PAR 3 | 0.28 | 0.11 | **.010** | .030 |
|  | | INT 3 – PAR 4 | 0.28 | 0.11 | **.010** | .031 |
|  | | PAR 1 – INT 2 | 0.01 | <0.01 | **<.001** | .055 |
|  | | PAR 2 – INT 3 | 0.01 | <0.01 | **<.001** | .060 |
|  | | PAR 3 – INT 4 | 0.01 | <0.01 | **<.001** | .067 |
|  | | EXT 1 – PAR 2 | 0.13 | 0.07 | .056 | .025 |
|  | | EXT 2 – PAR 3 | 0.13 | 0.07 | .056 | .021 |
|  | | EXT 3 – PAR 4 | 0.13 | 0.07 | .056 | .022 |
|  | | PAR 1 – EXT 2 | <0.01 | <0.01 | **.018** | .021 |
|  | | PAR 2 – EXT 3 | <0.01 | <0.01 | **.018** | .022 |
|  | | PAR 3 – EXT 4 | <0.01 | <0.01 | **.018** | .025 |
| Indirect effects * | | |  |  |  |  |
|  | | PAR 1 – INT 2 – PAR 3 | <0.01 | <0.01 | **.028** | .002 |
|  | | PAR 2 – INT 3 – PAR 4 | <0.01 | <0.01 | **.028** | .002 |
|  | | INT 1 – PAR 2 – INT 3 | <0.01 | <0.01 | **.028** | .002 |
|  | | INT 2 – PAR 3 – INT 4 | <0.01 | <0.01 | **.028** | .002 |
|  | | PAR 1 – EXT2 - INT 3 – PAR 4 | <0.01 | <0.01 | .209 | <.001 |
|  | | EXT 1 - INT 2 – PAR 3 – EXT 4 | <0.01 | <0.01 | .209 | <.001 |

*Note.* INT = SDQ internalising symptoms. EXT = SDQ externalising symptoms. PAR = DASS score. Numbers 1 to 4 refer to data collection time points (1 = May’20, 2 = Sep’20, 3 = Jan’21, 4 = May’21). **Bold** indicates significant pathways. * Indirect effects were assessed using bootstrapping method with 5,000 samples.

#### Direct effects in the age-moderated CLPM

For primary school aged children, all cross-lagged pathways could be constrained to be time-invariant, indicating stable associations between offspring internalising symptoms, offspring externalising symptoms, and parent internalising symptoms over time. We found that, similarly to RI-CLPM, parent internalising symptoms predicted higher subsequent offspring internalising (medium effects; β: .057 - .071) symptom scores. Yet, in contrast to RI-CLPM, parent internalising symptoms also predicted later offspring’s externalising (small effects; β: .025 - .031) symptom scores. Offspring symptoms did not significantly predict parent symptoms over time, in line with RI-CLPM.

For secondary school aged children, only bidirectional paths between parent internalising and offspring internalising symptoms and offspring-driven paths from offspring externalising to parent internalising symptoms could be constrained to be time-invariant. In contrast to the main CLPM or primary school aged group, constraining of the cross-lagged paths from parent internalising to offspring externalising symptoms to be time-invariant led to significantly worse model fit (Table S14), suggesting that these relationships changed between waves. Specifically, there was a significant bidirectional relationship between parent internalising symptoms and offspring internalising symptoms at all time points with medium effect sizes (β: .040 - .061). There was also a consistent and significant medium size offspring-driven effect (β = .039 – .045) from externalising symptoms to parent internalising symptoms. Large parent-driven effects on offspring externalising symptoms existed over two cross-lagged time periods. Specifically, higher parent internalising symptoms in May 2020 predicted higher offspring externalising symptoms in September 2020 (β = .133) and higher parent internalising symptoms in September 2020 predicted higher offspring externalising symptoms in January 2021 (β = .256). This differed substantially from the RI-CLPM analysis, where consistent bidirectional relationships were found between parent internalising and offspring externalising symptoms, instead.

**Table S15**

*Model selection and fit indices for multi-group CLPM for age*

| Model | Constraints added | *df* | AIC | BIC | CFI | TLI | RMSEA | SRMR | Δχ² | Δ*p* |
| --- | --- | --- | --- | --- | --- | --- | --- | --- | --- | --- |
| A1 | Base model (no constraints) | 144 | 108419 | 109249 | .999 | .995 | .020 | .009 | - | - |
| A2 | A1 + Cross-lagged constraints within and between groups | 114 | 108405 | 109062 | .997 | .995 | .021 | .018 | 46.39 | .029 |
| A3 | A1 + Cross-lagged constraints within groups | 120 | 108413 | 109105 | .997 | .994 | .022 | .017 | 42.32 | .012 |
| **A4** | **A1 + Cross-lagged constraints within primary school aged group only** | **132** | **108402** | **109163** | **.999** | **.997** | **.015** | **.012** | **7.42** | **.829** |
| **A5** | **A4 + INT on PAR constraints within secondary school aged group** | **130** | **108404** | **109153** | **.999** | **.997** | **.017** | **.012** | **5.50** | **.064** |
| **A6** | **A5 + PAR on INT constraints within secondary school aged group** | **128** | **108402** | **109139** | **.999** | **.997** | **.016** | **.013** | **1.95** | **.377** |
| A7 | A6 + INT on EXT constraints within secondary school aged group | 126 | 108406 | 109132 | .998 | .996 | .019 | .015 | 8.37 | .015 |
| **A8** | **A6 + EXT on INT constraints within secondary school aged group** | **126** | **108401** | **109127** | **.999** | **.997** | **.016** | **.013** | **3.00** | **.224** |
| A9 | A8 + EXT on PAR constraints within secondary school aged group | 124 | 108411 | 109126 | .998 | .995 | .021 | .015 | 14.56 | .001 |
| **A10** | **A8 + PAR on EXT constraints within secondary school aged group** | **124** | **108399** | **109114** | **.999** | **.997** | **.017** | **.014** | **2.71** | **.259** |

*Note.* INT = SDQ internalising symptoms. EXT = SDQ externalising symptoms. PAR = DASS score. A1 to A10 indicate the order of models for the χ² difference testing. **Bold** indicates acceptable model constraints.

**Table S16**

*Auto-regressive, concurrent, and cross-lagged paths in the age-moderated CLPM model*

| Parameters | | | | Primary school aged (4-10 years old) | | | |  | Secondary school aged (11-16 years old) | | | |
| --- | --- | --- | --- | --- | --- | --- | --- | --- | --- | --- | --- | --- |
|  |  |  |  | *B* | *SE* | *p* | β |  | *B* | *SE* | *p* | β |
| Concurrent paths | | | |  |  |  |  |  |  |  |  |  |
|  | | | INT 1 – EXT 1 | 4.22 | 0.33 | **<.001** | .430 |  | 5.80 | 0.51 | **<.001** | .502 |
|  | | | INT 2 – EXT 2 | 1.40 | 0.16 | **<.001** | .335 |  | 0.97 | 0.17 | **<.001** | .291 |
|  | | | INT 3 – EXT 3 | 1.00 | 0.12 | **<.001** | .301 |  | 0.91 | 0.14 | **.001** | .303 |
|  | | | INT 4 – EXT 4 | 0.90 | 0.12 | **<.001** | .280 |  | 1.10 | 0.15 | **<.001** | .386 |
|  | | | INT 1 – PAR 1 | 16.92 | 1.63 | **<.001** | .351 |  | 20.09 | 2.34 | **<.001** | .371 |
|  | | | INT 2 – PAR 2 | 5.52 | 0.93 | **<.001** | .226 |  | 6.77 | 1.25 | **<.001** | .276 |
|  | | | INT 3 – PAR 3 | 6.73 | 0.95 | **<.001** | .262 |  | 3.57 | 1.09 | **.001** | .152 |
|  | | | INT 4 – PAR 4 | 4.56 | 0.86 | **<.001** | .199 |  | 4.65 | 0.98 | **<.001** | .386 |
|  | | | PAR 1 – EXT 1 | 26.97 | 2.52 | **<.001** | .358 |  | 22.95 | 3.40 | **<.001** | .282 |
|  | | | PAR 2 – EXT 2 | 6.13 | 1.30 | **<.001** | .182 |  | 4.73 | 1.44 | **.001** | .175 |
|  | | | PAR 3 – EXT 3 | 6.04 | 1.27 | **<.001** | .175 |  | 4.52 | 1.33 | **.001** | .159 |
|  | | | PAR 4 – EXT 4 | 6.33 | 1.07 | **<.001** | .227 |  | 4.62 | 1.29 | **<.001** | .176 |
| Auto-regressive paths | | | |  |  |  |  |  |  |  |  |  |
|  | Single-wave | | |  |  |  |  |  |  |  |  |  |
|  | | | INT 1 – INT 2 | 0.68 | 0.02 | **<.001** | .678 |  | 0.72 | 0.03 | **<.001** | .718 |
|  | | | INT 2 – INT 3 | 0.45 | 0.03 | **<.001** | .452 |  | 0.50 | 0.05 | **<.001** | .509 |
|  | | | INT 3 – INT 4 | 0.37 | 0.04 | **<.001** | .356 |  | 0.41 | 0.04 | **<.001** | .399 |
|  | | | EXT 1 – EXT 2 | 0.80 | 0.02 | **<.001** | .780 |  | 0.79 | 0.02 | **<.001** | .830 |
|  | | | EXT 2 – EXT 3 | 0.56 | 0.03 | **<.001** | .559 |  | 0.33 | 0.05 | **<.001** | .505 |
|  | | | EXT 3 – EXT 4 | 0.46 | 0.04 | **<.001** | .456 |  | 0.49 | 0.05 | **<.001** | .469 |
|  | | | PAR 1 – PAR 2 | 0.76 | 0.03 | **<.001** | .714 |  | 0.83 | 0.03 | **<.001** | .737 |
|  | | | PAR 2 – PAR 3 | 0.64 | 0.05 | **<.001** | .529 |  | 0.48 | 0.06 | **<.001** | .450 |
|  | | | PAR 3 – PAR 4 | 0.29 | 0.03 | **<.001** | .317 |  | 0.40 | 0.05 | **<.001** | .398 |
|  | | Two-wave | |  |  |  |  |  |  |  |  |  |
|  | | | INT 1 – INT 3 | 0.33 | 0.04 | **<.001** | .337 |  | 0.35 | 0.05 | **<.001** | .350 |
|  | | | INT 2 – INT 4 | 0.38 | 0.04 | **<.001** | .362 |  | 0.28 | 0.05 | **<.001** | .274 |
|  | | | EXT 1 – EXT 3 | 0.32 | 0.04 | **<.001** | .313 |  | 0.33 | 0.05 | **<.001** | .353 |
|  | | | EXT 2 – EXT 4 | 0.33 | 0.04 | **<.001** | .325 |  | 0.33 | 0.07 | **<.001** | .327 |
|  | | | PAR 1 – PAR 3 | 0.33 | 0.06 | **<.001** | .254 |  | 0.41 | 0.08 | **<.001** | .340 |
|  | | | PAR 2 – PAR 4 | 0.34 | 0.05 | **<.001** | .311 |  | 0.48 | 0.07 | **<.001** | .446 |
|  | | Three-wave | |  |  |  |  |  |  |  |  |  |
|  | | | INT 1 – INT 4 | 0.09 | 0.04 | **.039** | .086 |  | 0.22 | 0.05 | **<.001** | .219 |
|  | | | EXT 1 – EXT 4 | 0.13 | 0.04 | **.002** | .121 |  | 0.11 | 0.07 | .117 | .112 |
|  | | | PAR 1 – PAR 4 | 0.27 | 0.06 | **<.001** | .229 |  | 0.01 | 0.09 | .914 | .340 |
| Cross-lagged paths | | | | | | | |  |  |  |  |  |
|  | | | INT 1 – EXT 2 | 0.04 | 0.02 | **.036** | .027 |  | 0.06 | 0.02 | **.011** | .040 |
|  | | | INT 2 – EXT 3 | 0.04 | 0.02 | **.036** | .027 |  | 0.06 | 0.02 | **.011** | .041 |
|  | | | INT 3 – EXT 4 | 0.04 | 0.02 | **.036** | .026 |  | 0.06 | 0.02 | **.011** | .039 |
|  | | | EXT 1 – INT 2 | 0.02 | 0.01 | **.019** | .035 |  | 0.05 | 0.02 | .**027** | .071 |
|  | | | EXT 2 – INT 3 | 0.02 | 0.01 | **.019** | .036 |  | -0.03 | 0.02 | .198 | -.037 |
|  | | | EXT 3 – INT 4 | 0.02 | 0.01 | **.019** | .034 |  | 0.02 | 0.02 | .451 | .022 |
|  | | | INT 1 – PAR 2 | 0.22 | 0.14 | .126 | .027 |  | 0.35 | 0.16 | **.035** | .043 |
|  | | | INT 2 – PAR 3 | 0.22 | 0.14 | .126 | .022 |  | 0.35 | 0.16 | **.035** | .041 |
|  | | | INT 3 – PAR 4 | 0.22 | 0.14 | .126 | .024 |  | 0.35 | 0.16 | **.035** | .040 |
|  | | | PAR 1 – INT 2 | 0.01 | <0.01 | **<.001** | .057 |  | 0.01 | <0.01 | **.001** | .052 |
|  | | | PAR 2 – INT 3 | 0.01 | <0.01 | **<.001** | .061 |  | 0.01 | <0.01 | **.001** | .059 |
|  | | | PAR 3 – INT 4 | 0.01 | <0.01 | **<.001** | .071 |  | 0.01 | <0.01 | **.001** | .061 |
|  | | | EXT 1 – PAR 2 | 0.01 | 0.01 | .390 | .014 |  | 0.24 | 0.11 | **.031** | .045 |
|  | | | EXT 2 – PAR 3 | 0.01 | 0.01 | .390 | .012 |  | 0.24 | 0.11 | **.031** | .040 |
|  | | | EXT 3 – PAR 4 | 0.01 | 0.01 | .390 | .013 |  | 0.24 | 0.11 | **.031** | .039 |
|  | | | PAR 1 – EXT 2 | 0.01 | <0.01 | **.027** | .025 |  | 0.01 | 0.01 | **.004** | .133 |
|  | | | PAR 2 – EXT 3 | 0.01 | <0.01 | **.027** | .026 |  | 0.01 | <0.01 | **.023** | .256 |
|  | | | PAR 3 – EXT 4 | 0.01 | <0.01 | **.027** | .031 |  | -0.01 | <0.01 | .059 | .258 |
| *Indirect effects ** | | | |  |  |  |  |  |  |  |  |  |
|  | | | PAR 1 – EXT 2 – PAR 3 | <0.01 | <0.01 | .459 | <.001 |  | <0.01 | <0.01 | .107 | .003 |
|  | | | PAR 2 – EXT 3 – PAR 4 | <0.01 | <0.01 | .459 | <.001 |  | <0.01 | <0.01 | .107 | .002 |
|  | | | EXT 1 – PAR 2 – EXT 3 | <0.01 | <0.01 | .459 | <.001 |  | <0.01 | <0.01 | .172 | .002 |
|  | | | EXT 2 – PAR 3 – EXT 4 | <0.01 | <0.01 | .459 | <.001 |  | -<0.01 | <0.01 | .198 | -.002 |
|  | | | PAR 1 – INT 2 – PAR 3 | <0.01 | <0.01 | .203 | .001 |  | <0.01 | <0.01 | .071 | .002 |
|  | | | PAR 2 – INT 3 – PAR 4 | <0.01 | <0.01 | .203 | .001 |  | <0.01 | <0.01 | .071 | .002 |
|  | | | INT 1 – PAR 2 – INT 3 | <0.01 | <0.01 | .203 | .002 |  | <0.01 | <0.01 | .071 | .003 |
|  | | | INT 2 – PAR 3 – INT 4 | <0.01 | <0.01 | .203 | .002 |  | <0.01 | <0.01 | .071 | .002 |

*Note.* INT = SDQ internalising symptoms. EXT = SDQ externalising symptoms. PAR = DASS score. Numbers 1 to 4 refer to data collection time points (1 = May’20, 2 = Sep’20, 3 = Jan’21, 4 = May’21). **Bold** indicates significant pathways. * Indirect effects were assessed using bootstrapping method with 5,000 samples.

### Moderation by Gender

Similarly to RI-CLPM, associations between parent internalising, offspring internalising and offspring externalising symptoms were not significantly moderated by offspring gender (Table S16). The group-constrained model did not significantly reduce model fit in comparison to the unconstrained model in which all parameters could vary between groups (Δχ *^2^* (30) = 25.27, *p* = .712), suggesting that none of the pathways varied by gender.

### Indirect effects

In contrast to RI-CLPM, transactionality in the traditional CLPM models can only be evidenced via the presence if indirect stability paths due to confounding of within- and between- individual differences in direct cross-lagged paths. Thus, we explored whether any indirect pathways were involved in associations between parent and offspring symptoms in both the main CLPM (Table S13) and the age-moderated model (Table S14). In the main CLPM model, significant indirect effects occurred over time from earlier to later offspring internalising symptoms via parent internalising symptoms, and from earlier to later parent internalising symptoms via offspring internalising symptoms, suggesting sustained transactionality between parent and offspring internalising symptoms. However, these indirect effects were very small (β = .002). In the age-moderated model, none of the indirect paths reached statistical signiﬁcance (β: -.002 – .003) when the bootstrapping method was applied, similarly to the indirect pathways in the main RI-CLPM. Taken together, this suggests that parent-offspring mental health symptom exchange is still transactional in nature (i.e., changes in one bidirectionally relate to changes in the other in RI-CLPM above and beyond the between-individual differences) but these transactional effects might not be sustained longer term (i.e., indirect stability effects are weak or not present even when examined in less conservative CLMP models).

**Table S17**

*Model selection and fit indices for multi-group CLPM for gender*

| Model | Constraints added | *df* | AIC | BIC | CFI | TLI | RMSEA | SRMR | Δχ² | Δ*p* |
| --- | --- | --- | --- | --- | --- | --- | --- | --- | --- | --- |
| G1 | Base model (no constraints) | 144 | 108480 | 109309 | .999 | .995 | .020 | .009 | - | - |
| **G2** | **G1 + Cross-lagged constraints within and between groups** | **114** | **108445** | **109102** | **.999** | **.998** | **.013** | **.014** | **25.27** | **.712** |

*Note.* INT = SDQ internalising symptoms. EXT = SDQ externalising symptoms. PAR = DASS score. A1 to A10 indicate the order of models for the χ² difference testing. **Bold** indicates acceptable model constraints.
